# Spatial isolation and environmental factors drive distinct bacterial and archaeal communities in different types of petroleum reservoirs in China

**DOI:** 10.1038/srep20174

**Published:** 2016-02-03

**Authors:** Peike Gao, Huimei Tian, Yansen Wang, Yanshu Li, Yan Li, Jinxia Xie, Bing Zeng, Jiefang Zhou, Guoqiang Li, Ting Ma

**Affiliations:** 1 College of Life Sciences, Nankai University, Tianjin 300071, P.R. China; 2 Key Laboratory of Molecular Microbiology and Technology, Ministry of Education, Tianjin 300071, P.R. China

## Abstract

To investigate the spatial distribution of microbial communities and their drivers in petroleum reservoir environments, we performed pyrosequencing of microbial partial 16S rRNA, derived from 20 geographically separated water-flooding reservoirs, and two reservoirs that had not been flooded, in China. The results indicated that distinct underground microbial communities inhabited the different reservoirs. Compared with the bacteria, archaeal alpha-diversity was not strongly correlated with the environmental variables. The variation of the bacterial and archaeal community compositions was affected synthetically, by the mining patterns, spatial isolation, reservoir temperature, salinity and pH of the formation brine. The environmental factors explained 64.22% and 78.26% of the total variance for the bacterial and archaeal communities, respectively. Despite the diverse community compositions, shared populations (48 bacterial and 18 archaeal genera) were found and were dominant in most of the oilfields. Potential indigenous microorganisms, including *Carboxydibrachium*, *Thermosinus*, and *Neptunomonas*, were only detected in a reservoir that had not been flooded with water. This study indicates that: 1) the environmental variation drives distinct microbial communities in different reservoirs; 2) compared with the archaea, the bacterial communities were highly heterogeneous within and among the reservoirs; and 3) despite the community variation, some microorganisms are dominant in multiple petroleum reservoirs.

Petroleum reservoirs represent unique environments deep under the Earth’s crust, with multiphase fluids containing oil, gas, and water. Petroleum reservoirs are composed of multiple oil-bearing strata, of low porosity and low permeability, and represent complex oligotrophic ecosystems. Nevertheless, these extreme environments can harbour diverse bacteria and archaea that exhibit strong adaptations to the oligotrophic and oxygen-deficient conditions^1^,^2^,^3^. These microorganisms play key roles in the Earth’s biogeochemical cycles. Studies of oil reservoir ecosystems have improved our understanding of these biogeochemical cycles^4^,^5^,^6^,^7^,^8^ and the mechanisms that microorganisms use to tolerate the extreme underground environments^9^. Some studies have enhanced the potential for biotechnological applications, in particular, the use of biological preservatives^10^,^11^,^12^,^13^ and microbial enhanced oil recovery^14^,^15^. Diverse microbial populations inhabiting these reservoirs have been revealed^16^,^17^,^18^,^19^,^20^,^21^,^22^,^23^. However, due to limited sample sources and methodological constraints, few studies have attempted to characterize the large-scale biogeographic distributions of microbial communities in oil reservoirs.

There is growing evidence that free-living microorganisms exhibit non-random distribution patterns across diverse habitats, at various temporal and spatial scales^24^,^25^,^26^,^27^,^28^,^29^,^30^. Environmental variables, such as day–night differences^31^, temperature^32^,^33^, salinity^34^, pH^24^,^35^, and nutrients^15^,^36^ appear to be the major determinants of microbial community composition. The impacts of geographical distance on structuring microbial assemblages have also been observed in several environments, particularly soil and marine environments^25^,^37^. However, the complex exploitation history and diverse physicochemical properties of oil reservoirs, and the low coverage of traditional detection methods, mean that there is limited knowledge regarding the microbial communities that are present and how they are influenced by spatial and environmental variables (relative to the equivalent knowledge for soil and marine environments)^38^,^39^. Previous studies that investigated the microbiology of oil reservoirs generally covered relatively small spatial scales. Therefore, the differences in microbial communities between different reservoir ecosystems, and the changes in the communities along environmental gradients, were difficult to elucidate.

Microbial distribution patterns across large spatial scales and their underlying driving mechanisms have become a focus in microbial ecological research. In this study, bacterial and archaeal communities from 20 geographically-separated water-flooding reservoirs, and two reservoirs that had not been flooded, in China, were analysed through 454-pyrosequencing of the 16S rRNA gene. Pyrosequencing generates thousands of short sequences representing different biota and significantly improves our ability to compare microbial populations in detail. The aim of this study was to determine the composition and spatial distribution of the bacterial and archaeal communities and determine the major environmental variables (mining patterns, spatial isolation, temperature, salinity, and pH) in shaping their composition and structure across a broad range of physical and geochemical reservoirs.

## Results

### Reservoir characteristics

**Figure 1:**
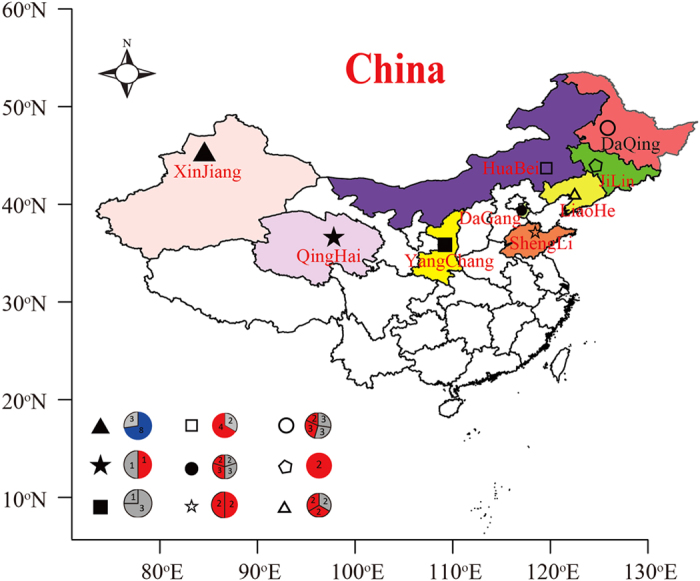
Locations of the sampling sites in the 22 geographically-separated reservoirs in China. The Arabic numerals in the pie charts represent the number of samples; the blue parts of the pie charts represent the reservoirs with temperatures of 22 °C; gray parts represent reservoirs with temperature of 34–45 °C; red parts represent reservoirs with temperatures of 46–73 °C. The map was produced using ‘R (i386 3.1.2)’, with the open source packages ‘maps’, ‘mapdata’, and ‘maptools’.

There were striking differences in both geographic location and environmental characteristics among the 22 oil reservoirs (Fig. 1, Table 1 and Supplementary Table S1). The temperature, salinity, and concentrations of ions in the formation brines were significantly different between the reservoirs within most of the oilfields. The distance between each of the reservoirs ranged from tens to thousands of kilometres. Even the reservoirs located the same oilfield generally contained different layers of oil-bearing strata underground. For instance, the DGD, DGX, DGK, and DGS reservoir blocks were all located in the DG oilfield, but there were large differences in the layers and the depths of the oil-bearing strata. The mining patterns and history of flooding among the reservoirs were also generally different (Fig. 1 and Table 1). All of the reservoir blocks except YC2 and LHJ had been flooded at some point in the past; the production well sampled in the YC2 reservoir had not previously been flooded with water when the samples were collected, while steam soaking techniques had been conducted in the production wells sampled within the LHJ reservoir block.

There was often variation in the physical properties observed within the same reservoir. In the XJL reservoir block, the oil-bearing strata were divided into three: S_7_^3−1,^ S_7_^3−2^ and S_7_^3−3^. The SLZ reservoir block included G3, G4, and G6 oil-bearing strata, with depths ranging from 1187 to 1304 m (Table 1). Furthermore, despite being located in the same oil-bearing stratum, the depth of the oil-bearing stratum could vary greatly, for example, 1162 to 2162 m, in DGD block (Table 1). The mining patterns, flooding history, and environmental variation suggest that the environmental variables in the reservoirs are complex and volatile, with patchy distributions; this implies that there may be complex microbial community compositions and structures in the reservoirs.

### Overall microbial community composition

After filtering the low quality reads and chimeras, an average of 4,261 high-quality bacterial and 2,428 archaeal sequences were obtained in each water sample (Supplementary Tables S2 and S3). These sequences were assigned into 11,195 bacterial OTUs and 3,288 archaeal OTUs (at a genetic distance of 3%), with an average of 312 bacterial OTUs and 159 archaeal OTUs in each sample. The number of OTUs, and the Chao 1, ACE, Shannon’s and Simpson’s indices are summarized in Supplementary Tables S2 and S3. The coverage of the sequencing libraries reached 95%, which could reflect the whole of the microbial community in each water sample. However, high numbers of rare OTUs that were represented by one sequence were detected in each water sample. These taxa accounted for approximately 50% of all the bacterial or archaeal OTUs detected, but only accounted for less than 10% of all the sequences in each sample (Supplementary Tables S2 and S3). These data indicate that the reservoirs harbour an abundance of rare microbial organisms from the underground environments.

**Figure 2:**
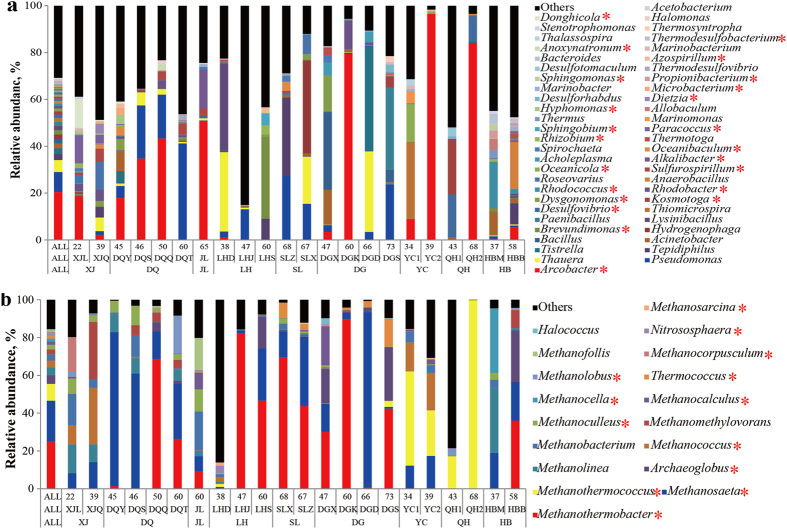
The relative proportion of dominant (a) bacterial and (b) archaeal genera in water samples obtained from the 22 reservoir blocks. Key: *represent genera for which their abundance was significantly different among oilfields, reservoir blocks, or reservoir temperatures (*P* < 0.05; see Supplementary Tables S8 and S10).

**Figure 3:**
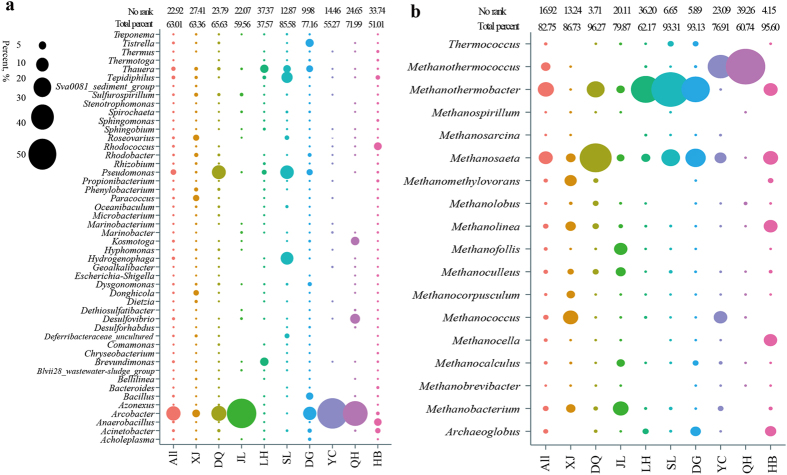
The (**a**) bacterial and (**b**) archaeal genera found in more than five of the nine oilfields. (**a**) 48 bacterial genera were detected in at least five of the nine oilfields, accounting for an average of 63.01% of the bacterial community in each oilfield; (**b**) 18 archaeal genera, accounting for 62.17–95.6% of the classified sequences, were observed in at least five of the nine oilfields.

At total of 34 bacterial phyla were identified; Proteobacteria, Firmicutes, Actinobacteria, Bacteroidetes, Thermotogae, and Synergistetes were detected, accounting for an average of 93.06% of the whole bacterial community, in every oilfield (Supplementary Fig. S1). At the class level, 53 taxa were identified. Of these, Epsilonproteobacteria, Alphaproteobacteria, Gammaproteobacteria, Betaproteobacteria, Deltaproteobacteria, Clostridia, Bacteroidia, Thermotogae, and Synergistia were detected, accounting for an average of 82.47% of the whole bacterial community, in every oilfield (Table 2). Alphaproteobacteria, Gammaproteobacteria, and Clostridia were detected in all of the 22 reservoir blocks, and accounted for an average of 2.02–68.49% of the bacterial community in each block (Table 2). At the genus level, a total of 380 phylotypes were defined. However, no taxa were detected in every reservoir block, or even across all oilfields. Each reservoir harboured certain unique microbial taxa with higher relative abundances (Fig. 2a). A total of 48 bacterial genera were detected in at least five of the nine oilfields, accounting for an average of 63.01% of the bacterial community in each oilfield (Fig. 3a). The most abundant phylotypes identified were *Arcobacter*, *Pseudomonas*, *Acinetobacter*, *Bacillus*, *Rhodococcus*, *Dietzia*, *Paracoccus*, *Marinobacterium*, *Rhizobium*, *Thauera*, *Rhodobacter*, *Brevundimonas*, *Desulfovibrio*, and *Desulforhabdus*; the rare genera found included *Tepidiphilus*, *Tistrella*, and *Kosmotoga*.

Compared to the bacterial communities, the archaeal communities did not vary very much at the phylum level. An average of 97.86% of the archaeal sequences identified at each oilfield belonged to Euryarchaeota (Supplementary Fig. S1). The archaeal sequences found belonged to the following classes: Methanomicrobia, Methanobacteria, Methanococci, Archaeoglobi, Thermoplasmata, Thermococci, Halobacteria, and Thermoprotei. Methanomicrobia, Methanobacteria, Methanococci, Thermoplasmata, and Halobacteria were detected in every oilfield, accounting for an average of 88.11% of the archaeal community in each oilfield (Table 2). Among them, *Methanomicrobia* and *Methanobacteria* were detected in all of the 22 reservoir blocks, accounting for an average of 70.52% of the archaeal community in each block. In total, 35 archaeal genera were detected in the 22 reservoir blocks (Fig. 2b). Among them, 18 genera, accounting for 62.17–95.6% of the sequences that could be classified, were found in at least five of the nine oilfields (Fig. 3b).

### Potential indigenous underground microbial populations

The microbial communities present in reservoirs that had not been flooded with water may reflect the indigenous underground populations. The LHJ reservoir block was a viscous oil reservoir that had been subjected to steam soaking for the exploration of the viscous oil. In this reservoir, Gammaproteobacteria and Alphaproteobacteria were dominant, and accounted for an average of 63.1% and 4.7% of the bacterial community, respectively. At the genus level, 83.4% of the sequences present could not be assigned to any taxa, using the RDP Classifier at the 80% threshold; this indicates that the reservoir harbours a large number of unclassified microbial taxa (Supplementary Fig. S2). In the reservoir, 27 genera were detected; among these, 24 genera were detected in at least some of the other reservoirs. However, three genera, including *Carboxydibrachium*, *Thermosinus*, and *Neptunomonas*, were detected specifically in LHJ, accounting for 0.02–0.11% of the bacterial community present. In terms of the archaeal community, four methane-producing archaea were detected in the LHJ reservoir. Among them, *Methanothermobacter* dominated the reservoir and accounted for 82.1% of the whole archaeal community.

The other reservoir that had not been flooded with water previous to sampling was the YC2 reservoir; this is an ultra-low permeability reservoir. Within this reservoir, 96.6% of the bacterial sequences were assigned to the class Epsilonproteobacteria. A total of nine genera, including *Arcobacter*, *Oceanicola*, *Marinobacterium*, *Guggenheimella*, *Geoalkalibacter*, *Marinobacter*, *Marinitoga*, *Sphingopyxis*, and *Rhodococcus*, were detected (Supplementary Fig. S2). Among them, *Arcobacter* accounted for 96.6% of the bacterial community present. Ten archaeal genera were detected in this reservoir. The dominant genera were the methane-producing *Methanococcus*, *Methanobacterium*, *Methanothermococcus*, *Methanocalculus*, and *Methanosaeta*.

### Regional distributions of the microbial populations

The bacterial and archaeal community α-diversity estimates, including the number of OTUs and Shannon’s index, exhibited associations within reservoir blocks. The LHJ, DGD, YC1, YC2, and QH2 reservoir blocks harboured low numbers of bacterial OTUs, and had lower Shannon’s indices (Supplementary Fig. S3). The QH2 reservoir had the smallest number of archaeal OTUs and the lowest Shannon’s index (Supplementary Fig. S3). However, the microbial community’s α-diversity (in particular, the archaeal) was not significantly correlated with the chemical parameters measured (*P < 0.05*; Supplementary Tables S5 and S6).

The microbial community composition and relative abundances of taxa were highly heterogeneous, even for samples retrieved from the same reservoir block (Fig. 2 and Table 2). The phylum Proteobacteria, containing the classes Alphaproteobacteria, Betaproteobacteria, Gammaproteobacteria, Deltaproteobacteria, and Epsilonproteobacteria, was detected in almost every reservoir. However, the abundances of these taxa differed greatly among the reservoirs (Table 2). For instance, significantly different relative abundances of Epsilonproteobacteria were found among the reservoir blocks (*P* < 0.05; Supplementary Table S7), whereby taxa in the class were dominant in the XJL, XJQ, DQY, DQS, DQQ, JL, DGK, YC1, YC2, and QH2 reservoir blocks, but only accounted for less than 1% of the bacterial communities in the LHJ, LHS, SLX, SLZ, DGD, DGS, QH1, and HBM reservoir blocks (Table 2). The dominant bacterial and archaeal taxa in each reservoir are summarized in Table 2 and Supplementary Tables S4, S7 and S9.

Although taxa of specific classes dominated multiple reservoir blocks, the dominant genera within the dominant class differed in some cases. In the Bacilli class, *Lysinibacillus* (37%) was dominant in the LHD block, *Bacillus* (33.18%) in the DGX block, *Paenibacillus* (35.04%) in the DGS block, and *Anaerobacillus* (20.07%) was dominant in the HBB block (Fig. 2a and Supplementary Table S4). This phenomenon may be partially explained by the reservoir temperatures among the four blocks: LHD was 38 °C; DGX, 47 °C; DGS, 73 °C; and the HBB block was 58 °C. A similar phenomenon was observed for the class Clostridia. This class was dominant in the QH1 and JL reservoir blocks, accounting for 41.20% and 32.46%, of the bacterial community in each block, respectively (Table 2). However, within Clostridia, *Alkalibacter* accounted for 16.66% of the bacterial community in the JL block, but was not detected in QH1 block; instead, *Desulfotomaculum* accounted for 3.66% in the QH1 block, and was not detected in the JL block (Fig. 2a). There were bacterial and archaeal taxa that had significantly different relative abundances between the reservoirs (*P* < 0.05; Supplementary Table S8 and S10); these data indicate that each reservoir harboured specific and dominant microbial taxa.

**Figure 4:**
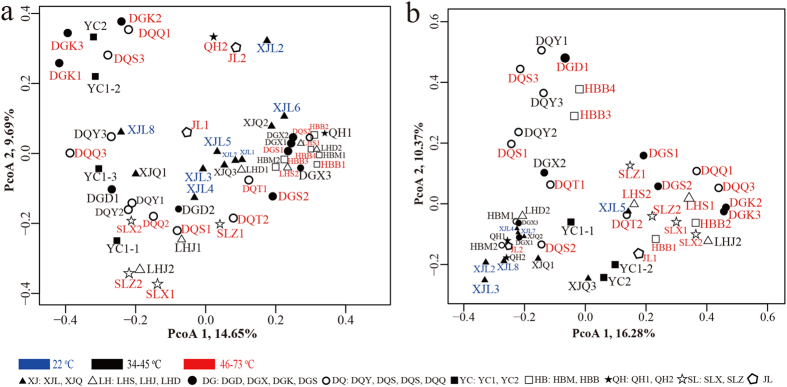
Principal coordinates analysis (PCoA) of the (**a**) bacterial and (**b**) archaeal communities, based on the UniFrac distance matrix generated from the rarefied taxon abundances and phylogenetic relationships. Points those are closer together on the ordination show communities that are more similar.

Through further investigation into the distribution of the reservoirs’ microbial communities, the cluster analysis and weighted UniFrac analysis performed, indicated that the distances between the communities within most of the reservoirs were significant (*P* < 0.001). Thus, it appears that distinct microbial communities did inhabit each reservoir. Although there are slight variances, predictable beta diversity patterns, for both the bacterial and archaeal communities, were observed. The bacterial and archaeal communities displayed high niche specificity; that is, the microbial communities from the same reservoir block formed clusters that differed from those of other reservoir blocks (Fig. 4 and Supplementary Fig. S4).

### Environmental variation and the microbial communities

As mentioned above, the LHJ, DGD, YC1, YC2, and QH2 reservoir blocks harbour low numbers of bacterial OTUs, and the QH2 reservoir has the smallest number of archaeal OTUs (Supplementary Fig. S3). Among these, steam-soaking techniques had previously been used in the sampled production wells in LHJ reservoir block; YC1 was an ultra-low permeability reservoir that was only flooded with water for 5 years; YC2 was an ultra-low permeability reservoir that had not been flooded with water previously; DGD was a high temperature and high pressure reservoir; and QH2 was a high temperature, hypersalinity, and high pressure reservoir. It appears that, the mining patterns, history of flooding, high temperatures, and hypersalinity exerted strong influences on the microbial communities in these reservoirs.

In accordance with these hypotheses, the relative abundance of 48 bacterial and 8 archaeal taxa exhibited strong correlations with reservoir temperature, and the formation brines’ pH, salinity, and/or ionic composition (*P* < 0.05; Supplementary Table S5 and S6). Some of the dominant bacterial and archaeal taxa (that have higher relative abundances) were significantly correlated with certain reservoir physicochemical parameters (Supplementary Fig. S5). *Paracoccus*, *Donghicola*, *Marinobacterium*, *Methanococcus*, *Methanocorpusculum*, and *Methanolinea* preferred to inhabit reservoirs with medium and low temperatures, while *Methanothermobacter* and *Thermococcus* were dominant in the reservoirs with higher temperatures. *Methanothermococcus* was found more frequently in reservoirs with high salinity. *Pseudomonas* was inversely correlated with the formation brine pH, while *Desulfuromonas* preferred to inhabit the more alkaline reservoirs.

**Figure 5:**
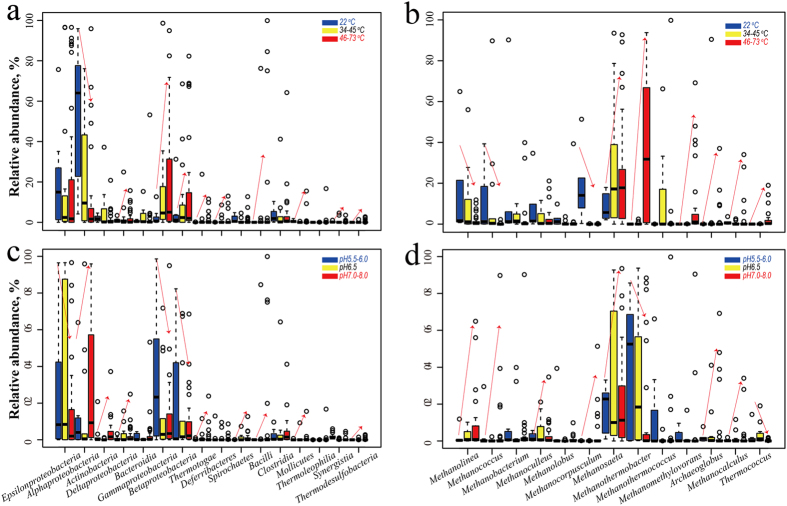
The distributions of dominant bacterial (**a**,**c**) and archaeal (**b,d**) populations against the temperature (**a**,**b**) and pH (**c,d**) gradients.

The dominant microbial populations were further clustered along with temperature and pH gradient, to highlight the populations that showed the most variability (Fig. 5 and Supplementary Fig. S6). At the class level, Epsilonproteobacteria were explicitly dominant in reservoirs with a pH of 5.5–6.5; Alphaproteobacteria were dominant in the low and medium temperature reservoirs, with a pH of 7.0–8.0; Actinobacteria were dominant in medium and high temperature reservoirs, with a pH of 7.0–8.0; Gammaproteobacteria and Betaproteobacteria were dominant in the medium and high temperature reservoirs, with with a pH of 5.5–6.5; and Deltaproteobacteria, Bacteroidia, Bacilli, Clostridia, Thermotogae, Mollicutes, Thermoleophilia, and Thermodesulfobacteria were clearly dominant in the medium–high temperature reservoirs (Fig. 5a,c). The genera *Rhodococcus*, *Paracoccus*, *Hyphomonas*, *Dietzia*, *Marinobacterium*, *Microbacterium*, and *Donghicola* were more dominant in the medium–low temperature reservoirs, while *Bacillus*, *Anaerobacillus*, *Thermotoga*, *Tepidiphilus*, *Tistrella*, *Thermodesulfovibrio*, *Thermodesulfobacterium*, *Thermus*, *Thermosyntropha*, and *Kosmotoga* had greater abundances in the medium–high temperature reservoirs (Supplementary Fig. S6). It’s worth mentioning that some taxa, including *Arcobacter*, *Pseudomonas*, *Acinetobacter*, *Sulfurospirillum*, *Rhizobium*, and *Sphingomonas* were universally detected in reservoirs with a wide range of temperatures. In terms of the archaea genera, *Methanolinea*, *Methanococcus*, *Methanoculleus*, *Methanolobus*, and *Methanocorpusculum* were dominant in the low and medium temperature reservoirs with alkaline environments; *Methanosaeta*, *Archaeoglobus*, and *Methanocalculus* dominated the medium and high temperature reservoirs with alkaline environments; while *Methanothermobacter* were more frequently detected in in medium and high temperature reservoirs with acidic environments (Fig. 5b,d).

**Figure 6:**
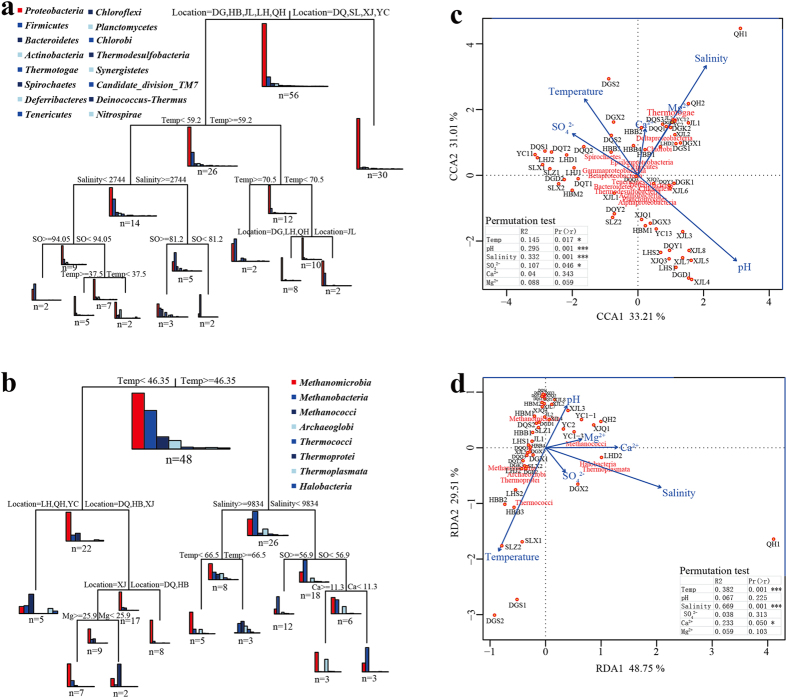
The relationship between the microbial community composition and various environmental conditions. The MRT analysis was performed to interpret the relationship of the relative abundance of dominant (**a**) bacterial and (**b**) archaeal lineages with the environmental variables measured. The canonical correspondence analysis (CCA) and redundancy analysis (RDA) used show the relationship between the environmental factors and the (**c**) bacterial and (**d**) archaeal communities.

A multivariate regression tree (MRT) analysis was performed to interpret the relationship between the relative abundances of dominant lineages and the main environmental variables (sampling location, reservoir temperature, and the formation brines’ pH, salinity, water type, and SO_4_^2−^, Ca^2+^ and Mg^2+^ concentrations) in a visualized tree. The trees collectively explained 60% and 64% of the variance observed in the relative abundances of the bacterial and archaeal taxa, respectively (Fig. 6a,b). In the bacterial MRT, the dominant lineages were first split by sampling location, which explained 16.1% of the variation in community structure. At the second node, the split was determined by temperature, which explained 9.0% of the variation. The communities were then split by salinity and temperature, accounting for 7.9% and 7.6% of the variation in the data, respectively. In terms of the archaeal MRT, the dominant lineages were first split by temperature, which explained 17.1% of the variation in community structure. The following left group was split by sampling location, while the right group was split by salinity, which explained 11.1% and 6.9% of the variation, respectively. The results suggest that spatial isolation, represented by sampling location, temperature, and the salinity of the formation brine, explained the variation in the microbial communities well.

To investigate the relationships between the microbial communities and the environmental variables of the petroleum reservoirs further, canonical correspondence analysis (CCA) and redundancy analysis (RDA) was conducted. The first two axes of the CCA and RDA analysis explained 64.22% and 78.26% of the total variance for the bacterial and archaeal communities, respectively (Fig. 6c,d). The Monte Carlo permutation test showed that temperature, salinity, concentrations of sulphate, and pH were significantly correlated with the changes in bacterial composition; temperature, salinity and calcium ion concentrations were significantly correlated with the changes in the archaeal communities (*P* < 0.05).

## Discussion

Microbial alpha-diversity, known as within-habitat diversity, reflects the number of species in a local homogeneous habitat. In petroleum reservoirs, both the bacterial and archaeal community α-diversities were influenced by the extreme environmental conditions; for example, steam-soaking (LHJ), non-water-flooding (YC2), high temperature, high pressure and hypersalinity (QH2) (Supplementary Fig. S3). However, species diversity, in particular that of the archaea, was not strongly correlated with the reservoir temperature and the chemistry of the formation brines (Supplementary Table S5 and S6). Actually, in previous studies, no relationship has been found between microbial α-diversity and either temperature or geographical distance, in other environments, such as in soil^38^ or acid mine drainage environments^24^. In terms of the microorganisms in reservoirs, studies have documented diverse microbial communities in Chinese reservoirs across a broad range of temperatures (22–73 °C), and these studies did not observe substantial differences in microbial α-diversity^18^,^19^,^40^. The phenomenon may be related to the fact that a large number of microbial populations, in particular thermophilic and thermotolerant organisms, are prevalent in medium and high temperature reservoirs, while other organisms prefer to inhabit medium and low temperature environments. Furthermore, most of the organisms found in these reservoirs are likely to possess exceptional survival abilities, and may be well adapted to, or tolerant of, the diverse reservoir environments.

Based on the phylogenetic analysis, more than 50 bacterial and 8 archaeal classes were identified across the 22 reservoirs. In accordance with previous research, Epsilonproteobacteria, Alphaproteobacteria, Gammaproteobacteria, Betaproteobacteria, Deltaproteobacteria, Bacilli, Clostridia, Actinobacteria, Bacteroidia, Thermotogae, Synergistia, Methanomicrobia, Methanobacteria, Methanococci, and Archaeoglobi were most frequently detected^16^,^19^,^21^,^40^,^41^,^42^. Despite the highly diverse microbial communities that inhabit petroleum reservoirs, some specific organisms were found to dominate the microbial communities across the different reservoirs. An increasing amount of studies have focused on the positive correlations between microbial community structure and function. For example, in marine environments^39^ and sewage treatment plants^43^; these studies have demonstrated that there are core microbes with specific functions across the different habitats.

In the petroleum reservoirs, 48 bacterial genera were detected, and found dominant, in the majority of the oilfields (Fig. 3a). These bacteria were mainly affiliated with the commonly reported hydrocarbon-degraders, surfactant-producers, nitrate-reducers, and sulphate-reducers. Among them, *Pseudomonas*, *Rhodococcus*, *Dietzia*, *Acinetobacter*, *Bacillus*, and *Paracoccus* are often aerobic organisms and are well known for their ability to degrade hydrocarbon or produce biosurfactants. Most of these populations are common in the microbial-enhanced oil recovery process^14^,^15^,^44^. *Pseudomonas*, *Brevundimonas*, *Hyphomonas*, *Arcobacter*, *Thauera*, *Rhizobium*, and *Rhodobacter* are able to reduce nitrate in either aerobic or anaerobic conditions. Among them, *Thauera* can anaerobically degrade aromatic compounds, and were the main contributor during the mitigation process of biological souring in oil reservoirs^3^,^13^. *Desulfovibrio* and *Desulforhabdus* are common sulfate-reducers in reservoir environments^1^,^18^.

In terms of the archaeal communities, 18 archaeal genera, which accounted for 62.17–95.6% of the classified sequences, were observed in most oilfields (Fig. 3b). Methylotrophic and hydrogenotrophic methanogens include: *Methanofollis*, *Methanocorpusculum*, *Methanocalculus*, *Methanothermococcus*, *Methanobacterium*, *Methanothermobacter*, *Methanococcus*, *Methanoculleus*, *Methanomethylovorans*, and *Methanolobus*^18^,^19^,^45^. These genera were found in abundance in the XJL, XJQ, DQS, DQQ, DQT, JL, LHJ, LHS, SLX, SLZ, DGX, DGK, DGS, YC1, YC2, QH1, QH2, and HBB reservoir blocks. *Methanosaeta*, which uses only acetate in methane production^45^, was dominant in the XJL, XJQ, DQY, DQS, DQQ, DQT, JL, LHS, SLX, SLZ, DGX, DGD, YC1, YC2, HBM, and HBB reservoir blocks. Therefore, the microbial organisms found across the different petroleum reservoirs may be closely related to the biogeochemical cycles that occur ubiquitously underground; in particular the carbon, nitrogen and sulphur cycles.

Microbial populations in reservoirs that had not previously been subjected to water flooding (LHJ and YC2) were analysed because they may reflect, to some degree, the indigenous populations before mining operations. Most of the taxa observed in the LHJ block were facultative bacteria and methanogens (Fig. 2 and Supplementary Table S4). Furthermore, three genera (*Carboxydibrachium*, *Thermosinus*, and *Neptunomonas*) were only detected in the LHJ reservoir. Species of the *Carboxydibrachium* and *Thermosinus* genera are anaerobic, thermophilic, and facultatively carboxydotrophic bacteria^46^. Populations of these genera can grow chemolithotrophically on CO, producing equimolar quantities of H_2_ and CO_2_. In the YC2 reservoir, nine bacterial genera were detected, with *Arcobacter* accounting for 96.6% of the bacterial community present. *Arcobacter* are found in an unusually wide range of habitats, including oil reservoirs and petroleum-contaminated groundwater^3^,^47^,^48^. Therefore, *Arcobacter* populations may mainly exist in fractures of the oil-bearing strata, and could have been introduced by the oil recovery processes. Furthermore, the permeability of the YC2 reservoir was only 0.5 md, which may seriously inhibit microbial migration and dispersion in the oil-bearing strata.

Although they give an indication, the results of this study do not provide sufficient reliable information of the indigenous microbial communities in these habitats. The introduction of exogenous microorganisms in injected water, and other sources of contamination that arise from the enhanced oil recovery processes, have made it increasingly difficult to determine whether a microorganism is indigenous. Interestingly, it seems that the reservoirs that had previously been flooded with water had high microbial diversity than the two reservoirs that had not. Furthermore, a large number of aerobes were found to coexist with the facultative and obligate anaerobes in the reservoirs. This phenomenon suggests that the oil exploitation processes may introduce exogenous microorganisms into reservoirs. These exogenous microbial populations may possess exceptional survival abilities and form new communities with the indigenous microorganisms, or they may remain in a dormant state after being introduced into the reservoir strata.

Each reservoir harboured microbial communities with distinct structures; the community composition and the relative abundance of microbial organisms were highly heterogeneous among the different reservoirs. Microbial assemblages in the reservoirs may have been affected by various environmental disturbances; mining patterns, geographical isolation, reservoir temperatures, and chemical composition of the formation brine were all found to be important. Many scientists have reported that microbial communities have a close relationship with their geographical position, in both contiguous and island habitats^49^,^50^,^51^. However, similar research of those communities in reservoir environments was scarce. Consistent with the other environments, the bacterial and archaeal communities in the reservoirs displayed high niche specificity. The microbial communities from the same reservoir block, and those from reservoir blocks located in the same oil-bearing strata were more similar, structurally. The MRT analyses revealed that the microbial communities in the reservoirs could be distinguished by most of the predefined factors assessed, including location (reservoir block), temperature, and the salinity of the formation brine. The CCA and RDA analyses also demonstrated the significant influences that temperature, salinity, and pH had on the microbial communities in the reservoirs. Temperature has previously been hypothesized as the main factor that influences microbial communities in water-flooding reservoirs^18^,^19^,^32^,^40^. pH was previously found to significantly influence soil microbial communities and their metabolisms^25^,^38^,^52^. Another study that found that the major environmental driver of microbial community composition in natural environments was salinity, rather than extremes of temperature, pH, or other physical and chemical factors^53^.

Diverse microbial community structures in the production wells from the same reservoir were also observed. The differences observed may be related to the isolation of the oil-bearing strata, the depth span of the reservoir strata^1^,^20^,^23^, and/or stochastic processes^28^,^54^. The spatial isolation and low permeability of reservoir strata may exert a significant influence on the community composition and structure in oil wells, even those located the same reservoir block. The stochastic processes of microbial community assembling and succession may also lead to the differences in the microbial communities^54^. As a result, we found that even in the same reservoir block, only a small number of microbial organisms were observed across the water samples from different production wells in the same reservoir. This phenomenon has also been documented by analysing the microbial communities derived from the wellhead and downhole of injection wells, and from their effective production wells nearby^20^.

This study revealed the similarities and differences of the microbial communities in the 22 geographically separated reservoirs, which had diverse physicochemical properties. The results indicate that distinct microbial communities inhabit different reservoirs, and that they are mainly driven by mining patterns, geographic isolation, reservoir temperature, and the salinity and pH of formation brine. The results expand our knowledge of the broad trends of microbial community distributions in petroleum reservoirs in China. They can be used to guide biotechnological applications in the reservoirs, in particular the biological preservative and microbial enhanced oil recovery. In future work, more comparative analyses, based on time-series sampling at a diverse array of reservoirs will be performed to better determine the community distributions along the reservoir environmental gradients.

## Methods

### Reservoir information

Samples were collected from 22 geographically separated reservoirs within nine oilfields across China (Fig. 1). The reservoirs, even those located in the same oilfield, represented a broad variety of physical and geochemical conditions, including differences in their exploitation history, temperature, and the salinity of the formation brine (Table 1). The DaQing (DQ), JiLin (JL), and LiaoHe (LH) oilfields are located in Northeast China; the ShengLi (SL), DaGang (DG), and HuaBei (HB) oilfields are located in Northern China; the YangChang (YC), QingHai (QH), and XinJiang (XJ) oilfields are located in Northwest China. Among them, the XJ, LH, SL, and HB oilfields each included two geographically isolated reservoir blocks, with different reservoir temperatures; the DG oilfield included three geographically isolated reservoir blocks, with different reservoir temperatures; and the DQ oilfield included four geographically isolated reservoir blocks, which could be classed into three groups, based on temperature. The temperatures ranged from 22 °C to 73 °C, while the salinity of the formation water in the reservoirs was 1,195–82,827 mg L^−1^. The samples were numbered with the name of reservoir block followed by Arabic numerals, such as XJL1, which represented the first water sample obtained from an oil well of Liuzhongqu reservoir block in XinJiang oilfield. The sampling oil wells of the same reservoir block were adjacent, located in a relatively closed site, and flooded by the same injected water. The ionic composition of the reservoirs’ formation waters varied greatly; detailed information of the ionic composition and other factors is shown in Supplementary Table S1.

### Samples collection and DNA extraction

Samples were collected between July 2011 and September 2012 from the wellheads of the production wells in each reservoir block. Sterilized plastic bottles (15 L) were completely filled, then immediately capped and sealed to avoid contamination and oxygen intrusion. The residual oil in each sample was firstly removed by heating the sample to 60 °C for 15 min and conducting phase separation in sterilized separatory funnels. Microbial cells were then collected from the 5 L water sample by centrifugation (12,000 × *g*) at 4 °C for 15 min, in a high-speed centrifuge (Beckman, USA). Total genomic DNA was extracted from the cell deposits using methods previously described^15^. The detailed process is described in the Supplementary text.

### PCR amplification and bar-coded pyrosequencing

The widely used universal primers, 27F (5′-AGA GTT TGA TCC TGG CTC AG-3′) and 533R (5′-TTA CCG CGG CTG CTG GCA C-3′), were used to amplify the bacterial 16S rRNA gene^55^; and the primers 344F (5′-ACG GGG YGC AGC AGG CGC GA-3′) and 915R (5′-GTG CTC CCC CGC CAA TTC CT-3′) were used for the archaeal 16S rRNA gene^56^. The primer sets have high coverage levels of bacterial and archaeal 16S RNA genes, respectively, and are the most effective primers for analyzing the diversity of microbial communities. Amplicon pyrosequencing was performed on a Roche Genome Sequencer GS FLX + platform at Majorbio Bio-Pharm Technology, Shanghai, China. The detailed PCR process and preparation steps for the sequencing of the amplicons is included in the Supplementary text.

The raw data generated were processed and analysed following the MOTHUR pipeline (http://www.mothur.org)^57^. Sequences with ambiguous bases and low quality scores (<25), those smaller than 200 bp, and sequence tags, chimeras and non-ribosomal sequences, were eliminated from the data sets. The remaining sequences were grouped into operational taxonomic units (OTUs) by setting a 0.03 distance limit (equivalent to 97% similarity). Representative sequences were aligned using NAST^58^. Taxonomic classification of the phylotypes was determined based on the Ribosomal Database Project, at the 80% confidence level^59^.

### Statistical analyses

The relative abundance (%) of individual taxa within each community was estimated by comparing the number of sequences assigned to the specific taxon versus the number of total sequences obtained for that sample. Rarefaction curves were produced based on the identified OTUs, then Shannon’s diversity index and non-parametric measures of species richness (Chao1 and ACE) were calculated for each sample using the MOTHUR pipeline. Finally, clustering analysis and PCoA, based on the UniFrac dissimilarity values, was performed, to interpret the relative similarity of the microbial communities from each sample site.

Linear correlations between the microbial alpha diversity, microbial abundance and environmental factors were examined using Pearson’s correlation analyses. Line charts and Boxplots were used to show the distribution of bacterial and archaeal populations along the environments gradients. Changes in microbial abundance in different reservoirs were compared using One Way Analysis of Variance (ANOVA) with Student-Newmnan-Keuls tests. An MRT analysis was performed using the package ‘mvpart’ within the ‘R’ statistical programming environment, to highlight the main relationships between the biological data and environmental variables^60^. The ‘1se’ cross-validation process was used to construct the multivariate regression tree. In addition, the correlations between the microbial communities and environmental factors were determined with Canonical correspondence analysis (CCA) and Redundancy analysis (RDA), using the ‘Vegan’ package within R (http://cran.r-project.org/web/packages/vegan). Statistical significance was assessed using the Monte Carlo permutation’s method, based on 999 permutations.

### Sequence accession numbers

The raw reads have been deposited in the National Center for Biotechnology Information (BioProject ID: PRJNA 271507, http://www.ncbi.nlm.nih.gov/bioproject/271507).

## Additional Information

**How to cite this article**: Gao, P. *et al.* Spatial isolation and environmental factors drive distinct bacterial and archaeal communities in different types of petroleum reservoirs in China. *Sci. Rep.* **6**, 20174; doi: 10.1038/srep20174 (2016).

## Supplementary Material

Supplementary Information

## Figures and Tables

**Table 1:** Characteristics of the 22 geographically-separated reservoirs in China.

Oil Field	Blocks	Flooding Date	Temp, °C	Depth, m	Pressure, MPa	Permeability, × 10^−3^ μm^2^	pH	#Salinity mg·L^−1^	#NO_3_^−^ mg·L^−1^	#PO_4_^3−^ mg·L^−1^	#SO_4_^2−^ mg·L^−1^	#Ca^2+^ mg·L^−1^	#Mg^2+^ mg·L^−1^
XinJiang (XJ)	Qizhongqu (XJQ)	1965	39	1088	15.0	274	7.0	11316.4	1.1	0.7	10.3	101.2	34.3
	Liuzhongqu (XJL)	1973	22	480	7.6	466	8.0	9630.1	3.6	4.0	106.1	110.8	52.7
DaQing (DQ)	Nanerqu (DQY)	1998	45	1116–1155	10.3	599	7.0	4892.0	12.3	16.2	81.1	31.5	60.2
	Beierqu (DQS)	2006	46	1064–1073	10.5	569	6.5	6157.4	3.4	1.9	46.2	22.1	5.2
	QingX-Wei1 (DQQ)	2006	50	1393–1411	11.5	141	6.0	8999.8	2.0	5.2	75.7	73.2	16.5
	Toutai-AO (DQT)	1994	60	1250–1520	15.6	3.8	5.5	12130.5	2.7	6.5	199.3	69.9	1.4
JiLin (JL)	Honggang (JL)	2005	65	2250	22.1	8	6.5	11098.8	4.4	10.3	118.2	20.5	9.6
LiaoHe (LH)	Shen84 (LHS)	1986	60	1800–2100	10.5	800	7	3609.7	4.4	5.5	47.9	11.5	9.3
	Jin45 (LHJ)	*1986	47	850–1060	4.5	1025	7	1665.4	0	0.9	120.3	18.5	9.3
	DXing35 (LHD)	1996	38	660–810	7.4	1350	7	1558.5	0.35	0.9	94.1	48.2	10.2
ShengLi (SL)	Gudaoxiqu (SLX)	1995	68	1187–1304	12.7	732	6	7000.5	ND	0.8	1783.5	132.4	75.4
	Zhongyiqu (SLZ)	1997	67	1198–1230	11.9	1196	6.5	5777.3	1.8	4.6	174.5	87.2	25.4
DaGang (DG)	Gangdong (DGD)	1968	66	1162–2162	15.4	872	6.5	3614.5	7.4	8.5	140.3	7.3	4.2
	Gangxi (DGX)	2002	47	1775–1790	17.5	230	7	9971.5	0.3	0.7	163.3	69.1	7.8
	Kongdian (DGK)	1972	60	1206–1343	13.2	7878	6.5	6755.0	1.3	5.7	92.3	65.9	32.8
	Shenvsi (DGS)	1984	73	1801–1980	12.0	468	6.5	22048.3	10.2	11.2	341.8	222.1	94.5
YanChang (YC)	Yangmahe (YC1)	2008	34	505–565	3.8	12.3	6	27991.5	15.8	19	1944.1	4354	487.8
	Yangmahe (YC2)	ΔNon	39	810	5.2	0.5	7	27965.6	22.6	19.2	2421.8	464.4	3952.0
QinHai (QH)	Yuejin (QH1)	2003	43	500–2000	13.0	210	7	82827.0	31.5	26.3	435.7	2293.0	516.7
	Yuexi (QH2)	2003	68	1380–1820	19.4	75	7	61082.0	19.5	23.6	55.9	2252.0	1314.0
HuaBei (HB)	Menggulin (HBM)	1989	37	750–830	7.5	675	7	1228.6	0.6	ND	9.1	17.1	5.0
	Ba19 (HBB)	2003	58	1500–1600	13.7	145	7	2585.5	0.5	1.7	41.7	46.0	5.6

^#^The concentration of ions is the average value of the water samples obtained from the same reservoir.

^*^Steam soaking techniques had been conducted in the LHJ reservoir block.

^Δ^The YC2 reservoir block had not previously been flooded with water when the samples were collected.

**Table 2:**
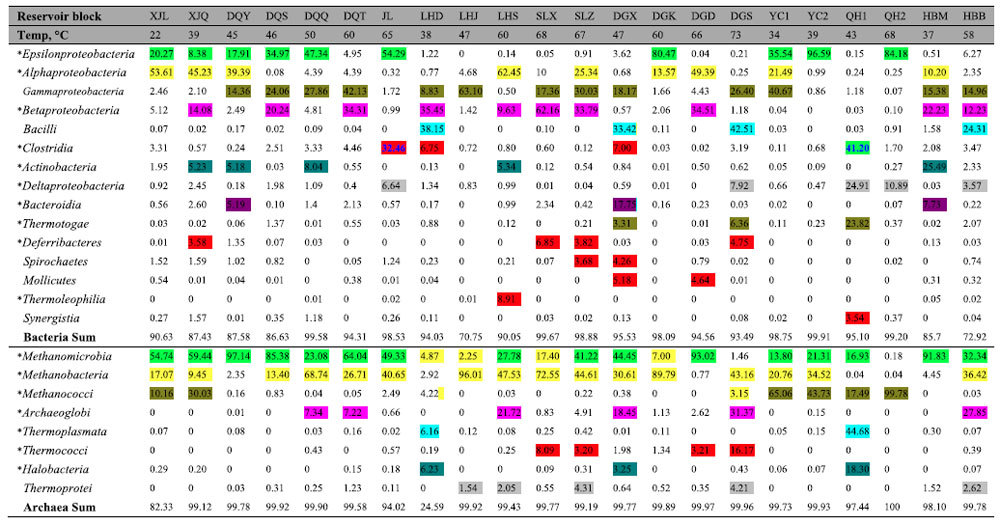
The dominant bacterial and archaeal taxa in the 22 geographically-separated oil reservoirs in China.

^*^The microbial populations are significantly different between oilfields, reservoirs, or reservoir temperatures (P<0.05; see Supplementary Tables S7 and S9).
