# Supplementary material for: Spatial isolation and environmental factors drive distinct bacterial and archaeal communities in different types of petroleum reservoirs in China
Source: Sci Rep. 2016 Feb 3;6:20174. doi: 10.1038/srep20174 (PMC4738313; doi:10.1038/srep20174)
Supplement: Supplementary Information [file srep20174-s1.doc]

**Supporting information**

Spatial isolation and environmental factors drive distinct bacterial and archaeal communities in different types of petroleum reservoirs in China

**Running Title:** Distinct microbial communities among reservoirs

**Authors**

Peike Gao^1^, Huimei Tian^1^, Yansen Wang^1^, Yanshu Li^1^, Yan Li^1^, Jinxia Xie^1^, Bing Zeng^1^, Jiefang Zhou^1^, Guoqiang Li^1,2^* & Ting Ma^1,2^*

**Affiliations**

^1^ College of Life Sciences, Nankai University, Tianjin 300071, P. R. China.

^2^ Key Laboratory of Molecular Microbiology and Technology, Ministry of Education, Tianjin 300071, P. R. China.

***Corresponding author**

T Ma. Mailing address: College of Life Sciences, Nankai University, Tianjin 300071, P. R. China. Tel/Fax: 86-22-23498185. E-mail: [tingma@nankai.edu.cn](mailto:tingma@nankai.edu.cn).

GQ Li. Mailing address: College of Life Sciences, Nankai University, Tianjin 300071, P. R. China. E-mail: [ligq@nankai.edu.cn](mailto:ligq@nankai.edu.cn).

**Supplemental Experimental Procedures**

**DNA extraction.** The cell deposits obtained were firstly resuspended with TE buffer (Tris 80 mM, EDTA 40 mM, pH 8.0) and then lysed using a mini bead-beater (BioSpec, USA) at 200 rpm for 2 min at room temperature with 0.1 mm glass beads. After bead beating, lysozyme was added (final concentration 1 mg/mL), and the samples were incubated at 37°C for 1 h. Then, 120 μL sodium dodecyl sulphate (20% SDS, W/V) was added and the samples were incubated at 65°C for 60 min. Total genomic DNA was then extracted from the suspension solution using an AxyPrepTM Genomic DNA miniprep kit (Axygen, USA) according to the manufacturer’s instructions. The extracted DNA was checked by agarose gel electrophoresis. The extracted DNA was stored at −80°C until further analysis.

**PCR amplification and bar-coded pyrosequencing.** The universal primers 27F (5’-AGA GTT TGA TCC TGG CTC AG-3’) and 533R (5’-TTA CCG CGG CTG CTG GCA C-3’) were used to amplify the bacterial 16S rRNA gene; and the primers 344F (5’-ACG GGG YGC AGC AGG CGC GA-3’) and 915R (5’-GTG CTC CCC CGC CAA TTC CT-3’) were used for the archaeal 16S rRNA gene. The primers 533R and 344F contained a linker for sequencing, and unique Roche multiplex identifiers (MID) to tag the PCR amplicons from each water sample. The PCR reactions were performed in triplicate with 10.5 µL sterile ddH_2_O, 4 µL 5-fold reaction buffer (TransStart® FastPfu Buffer, TransGen Biotech, China), 2 µL 2.5 mM dNTPs, 0.5 µL Pfu polymerase (TransStart® FastPfu DNA Polymerase, TransGen Biotech, China), 0.5 µL forward primer (10 μM), 0.5µL reverse primer (10 μM), and 2 µL sample DNA. To amplify the bacterial 16S rRNA, the samples were denatured for 2 min at 95°C, followed by 25 cycles of 94°C for 30 s, 50°C for 30 s, and 72°C for 30 s, then a final elongation step was performed at 72°C for 5 min. For the archaeal 16S rRNA, the samples were denatured for 2 min at 95°C, followed by 30 cycles of 94°C for 30 s, 50°C for 30 s, and 72°C for 30 s, then a final elongation step of 72°C for 5 min.

The PCR products were extracted using the PCR fragment recovery kit. The amplicon concentrations were measured with the QuantiFluor™ dsDNA System (Promega, USA). Then, the amplicons from each reaction mixture were pooled, in equimolar ratios based on their measured concentrations, and subjected to an emulsion PCR to generate the amplicon libraries. Amplicon pyrosequencing was performed on a Roche Genome Sequencer GS FLX+ platform at Majorbio Bio-Pharm Technology, Shanghai, China.

**Supplemental Figure Legends**

**Figure S1** The relative proportion of Bacterial (a) and archaeal (b) taxa at phylum level in the reservoir blocks.

**Figure S2** The relative proportion of dominant (a) bacterial and (b) archaeal genera in LHJ and YC2 reservoir blocks. Key:
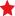
 represent the genera that were detected in at least some of the other reservoirs.

**Figure S3** Distribution of the observed species (a and c) and Shannon indexes (b and d) of bacterial (a and b) and archaeal (c and d) communities in the 22 geographically separated reservoirs.

**Figure S4** Clustering analysis of bacterial (a) and archaeal (b) communities from the 22 geographically separated reservoirs.

**Figure S5.** The line-charts show the partial dominant (a) bacterial genera and (b) archaeal genera that showed significant correlation with reservoir physicochemical parameters (*P < 0.05*; Supplementary Table S5 and S6).

**Figure S6** Distribution of bacterial populations at the genus level along with temperature gradient. This figure highlights the populations that showed the most variability along with temperature gradient. Key: XJL represents the low temperature reservoir (22°C); YC1, HBM, LHD, XJQ, YC2, QH1, and DQY represent reservoirs with temperatures 34°C–45°C; DQS, LHJ, DGX, DQQ, HBB, DQT, LHS, DGK, JL, SLZ, DGD, QH2, SLX, DGS represent reservoirs with temperatures 46°C–73°C. This figure highlights the populations that showed the most variability along the temperature gradient.

**Figure S1.** The relative proportion of Bacterial (a) and archaeal (b) taxa at phylum level in the reservoir blocks.

**Figure S2.** The relative proportion of dominant (a) bacterial and (b) archaeal genera in LHJ and YC2 reservoir blocks. Key:
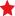
 represent the genera that were detected in at least some of the other reservoirs.

**Figure S3.** Distribution of the observed species (a and c) and Shannon indexes (b and d) of bacterial (a and b) and archaeal (c and d) communities in the 22 geographically separated reservoirs.

**Figure S4.** Clustering analysis of bacterial (a) and archaeal (b) communities from the 22 geographically separated reservoirs.

**Figure S5.** The line-charts show the partial dominant (a) bacterial genera and (b) archaeal genera that showed significant correlation with reservoir physicochemical parameters (*P < 0.05*; Supplementary Table S5 and S6).

**Figure S6.** Distribution of bacterial populations at the genus level along with temperature gradient. This figure highlights the populations that showed the most variability along with temperature gradient. Key: XJL represents the low temperature reservoir (22°C); YC1, HBM, LHD, XJQ, YC2, QH1, and DQY represent reservoirs with temperatures 34°C–45°C; DQS, LHJ, DGX, DQQ, HBB, DQT, LHS, DGK, JL, SLZ, DGD, QH2, SLX, DGS represent reservoirs with temperatures 46°C–73°C. This figure highlights the populations that showed the most variability along the temperature gradient.

**Supplemental Table Legends**

**Table S1** Characteristics of formation brines obtained from the 22 reservoirs in 9 oilfields, China

**Table S2** **and S3** Sequencing depth and alpha diversity of the bacterial and archaeal communities in each reservoir

**Table S4** The dominant bacterial genera in the 22 reservoir blocks, China

**Table S5 and S6** Pearson correlation coefficients of bacterial and archaeal α-diversity metrics and population abundance with the reservoir physicochemical parameters

**Table S7 to S10** One-Way ANOVA of the abundance of bacterial and archaeal taxa between the oilfields, reservoirs, reservoirs within the same oilfield, and temperatures

**Table S1** Characteristics of formation brines obtained from the 22 reservoirs in 9 oilfields, China

| Oil field | Sample ID | Temp | pH | Salinity | NO_3_^-^ | PO_4_^3-^ | SO_4_^2-^ | Ca^2+^ | Mg^2+^ | Water |
| --- | --- | --- | --- | --- | --- | --- | --- | --- | --- | --- |
| XinJiang  (XJ) | XJQ1 | 39 | 7 | 10916.1 | 1.4 | 2.1 | 11.9 | 17.4 | 13.6 | NaHCO_3_ |
|  | XJQ2 | 39 | 7 | 11645.5 | 0.9 | 0.2 | 13 | 151.8 | 58 | NaHCO_3_ |
|  | XJQ3 | 39 | 7 | 11387.5 | 1.1 | 0 | 5.9 | 134.3 | 31.2 | NaHCO_3_ |
|  | XJL1 | 22 | 8 | 10101.5 | 2.15 | 3.61 | 71.6 | 91.5 | 44.7 | NaHCO_3_ |
|  | XJL2 | 22 | 8 | 10687.3 | 6.3 | 2.9 | 61.5 | 116.2 | 64.5 | NaHCO_3_ |
|  | XJL3 | 22 | 8 | 9228.3 | 0 | 3.9 | 184.1 | 142.1 | 20.6 | NaHCO_3_ |
|  | XJL4 | 22 | 8 | 10040.9 | 3.7 | 2.9 | 126.8 | 117.5 | 36.5 | NaHCO_3_ |
|  | XJL5 | 22 | 8 | 8113.2 | 0.8 | 1.8 | 92.3 | 103.3 | 61.6 | NaHCO_3_ |
|  | XJL6 | 22 | 8 | 8997.6 | 4.6 | 6.8 | 61.6 | 86.9 | 75.1 | NaHCO_3_ |
|  | XJL7 | 22 | 8 | 9710.5 | 0 | 0 | 89.2 | 99.7 | 36.9 | NaHCO_3_ |
|  | XJL8 | 22 | 8 | 10161.2 | 11.3 | 9.8 | 161.5 | 128.8 | 81.6 | NaHCO_3_ |
| DaQing  (DQ) | DQY1 | 45 | 7 | 4673.9 | 11.9 | 21.6 | 126.3 | 11.7 | 42.5 | NaHCO_3_ |
|  | DQY2 | 45 | 7 | 4816.5 | 9.8 | 8.6 | 36.9 | 36.8 | 56.9 | NaHCO_3_ |
|  | DQY3 | 45 | 7 | 5185.6 | 15.2 | 18.4 | 80.2 | 46.1 | 81.2 | NaHCO_3_ |
|  | DQS1 | 46 | 6.5 | 6030.6 | 3.2 | 0 | 42.6 | 16.8 | 5.9 | NaHCO_3_ |
|  | DQS2 | 46 | 6.5 | 6125.1 | 6.9 | 5.8 | 67.2 | 38.3 | 7.1 | NaHCO_3_ |
|  | DQS3 | 46 | 6.5 | 6316.5 | 0 | 0 | 28.7 | 11.2 | 2.6 | NaHCO_3_ |
|  | DQQ1 | 50 | 6 | 8511.7 | 1.3 | 0 | 77.1 | 68.2 | 5.2 | NaHCO_3_ |
|  | DQQ2 | 50 | 6 | 9520.1 | 4.6 | 8.9 | 28.6 | 91.8 | 41.5 | NaHCO_3_ |
|  | DQQ3 | 50 | 6 | 8967.6 | 0 | 6.8 | 121.5 | 59.7 | 2.7 | NaHCO_3_ |
|  | DQT1 | 60 | 5.5 | 12831 | 2.8 | 5.1 | 216.9 | 78.5 | 2 | NaHCO_3_ |
|  | DQT2 | 60 | 5.5 | 11430 | 2.6 | 7.9 | 181.7 | 61.3 | 0.8 | NaHCO_3_ |
| JiLin  (JL) | JL1 | 65 | 6.5 | 6023.2 | 0 | 3.9 | 89.9 | 17.4 | 8.6 | NaHCO_3_ |
|  | JL2 | 65 | 6.5 | 16174.3 | 8.8 | 16.7 | 146.4 | 23.5 | 10.5 | NaHCO_3_ |
| LiaoHe  (LH) | LHS1 | 60 | 7 | 3532 | 5.6 | 4.8 | 28 | 9.9 | 9.4 | NaHCO_3_ |
|  | LHS2 | 60 | 7 | 3687.4 | 3.1 | 6.2 | 67.8 | 13.2 | 9.1 | NaHCO_3_ |
|  | LHJ1 | 47 | 7 | 1665.1 | 0 | 1.8 | 219.7 | 26.9 | 16.2 | NaHCO_3_ |
|  | LHJ2 | 47 | 7 | 1665.7 | 0 | 0 | 20.9 | 10 | 2.4 | NaHCO_3_ |
|  | LHD1 | 38 | 7 | 1617 | 0.7 | 0 | 86.6 | 35.1 | 10.5 | NaHCO_3_ |
|  | LHD2 | 38 | 7 | 1500 | 0 | 1.8 | 101.5 | 61.2 | 9.8 | NaHCO_3_ |
| ShengLi  (SL) | SLX1 | 72 | 6 | 8968.6 | 0 | 1.6 | 2719.6 | 96.9 | 86.9 | NaHCO_3_ |
|  | SLX2 | 65 | 6 | 5032.3 | 0 | 0 | 847.3 | 167.8 | 63.8 | NaHCO_3_ |
|  | SLZ1 | 64 | 6.5 | 6454.4 | 0 | 2.8 | 272 | 91.6 | 35.6 | NaHCO_3_ |
|  | SLZ2 | 70 | 6.5 | 5100.1 | 3.6 | 6.2 | 76.9 | 82.7 | 15.2 | NaHCO_3_ |
| DaGang  (DG) | DGD1 | 66 | 6.5 | 2704.3 | 6.6 | 10.1 | 21 | 12.6 | 3 | NaHCO_3_ |
|  | DGD2 | 66 | 6.5 | 4524.6 | 8.1 | 6.9 | 259.5 | 2 | 5.3 | NaHCO_3_ |
|  | DGX1 | 47 | 7 | 10246.9 | 0.9 | 0.2 | 35.6 | 35 | 5.5 | NaHCO_3_ |
|  | DGX2 | 47 | 7 | 9699.7 | 0 | 1.2 | 327.6 | 75.8 | 7.1 | NaHCO_3_ |
|  | DGX3 | 47 | 7 | 9967.9 | 0 | 0.8 | 126.8 | 96.5 | 10.8 | NaHCO_3_ |
|  | DGK1 | 60 | 6.5 | 6651 | 2.6 | 5.7 | 69.5 | 81.5 | 41.6 | NaHCO_3_ |
|  | DGK2 | 60 | 6.5 | 6457.6 | 1.3 | 6.1 | 82.4 | 66.7 | 33.8 | NaHCO_3_ |
|  | DGK3 | 60 | 6.5 | 7156.4 | 0 | 5.3 | 125 | 49.5 | 23 | NaHCO_3_ |
|  | DGS1 | 73 | 6.5 | 22225.2 | 11.6 | 9.8 | 136.5 | 168.2 | 98.9 | CaCl_2_ |
|  | DGS2 | 73 | 6.5 | 21871.3 | 8.7 | 12.6 | 547.1 | 276 | 90.1 | CaCl_2_ |
| YangChang  (YC) | YC1-1 | 34 | 6 | 31281.5 | 16.5 | 21.8 | 3583.6 | 4717 | 503.6 | CaCl_2_ |
|  | YC1-2 | 34 | 6 | 25155.9 | 9.6 | 18.9 | 1261.5 | 4066 | 462.9 | CaCl_2_ |
|  | YC1-3 | 34 | 6 | 27537 | 21.2 | 16.3 | 987.1 | 4279 | 496.9 | CaCl_2_ |
|  | YC2 | 39 | 7 | 27965.6 | 22.6 | 19.2 | 2421.8 | 464.4 | 3952 | CaCl_2_ |
| QingHai  (QH) | QH1 | 43 | 7 | 82827 | 31.5 | 26.3 | 435.7 | 2293 | 516.7 | CaCl_2_ |
|  | QH2 | 68 | 7 | 61082 | 19.5 | 23.6 | 55.9 | 2252 | 1314 | Na_2_SO_4_ |
| HuaBei  (HB) | HBM1 | 37 | 7 | 1262.1 | 1.26 | 0 | 13.5 | 16.8 | 0.8 | NaHCO_3_ |
|  | HBM2 | 37 | 7 | 1195 | 0 | 0 | 4.7 | 17.4 | 9.1 | NaHCO_3_ |
|  | HBB1 | 58 | 7 | 2891.3 | 0 | 1.35 | 12.1 | 3.6 | 1.4 | NaHCO_3_ |
|  | HBB2 | 58 | 7 | 2597.3 | 0 | 0 | 57.3 | 41.7 | 5 | NaHCO_3_ |
|  | HBB3 | 58 | 7 | 2431.1 | 2.18 | 1.57 | 40.6 | 64.7 | 6.9 | NaHCO_3_ |
|  | HBB4 | 58 | 7 | 2422.4 | 0 | 3.75 | 56.6 | 73.9 | 9.1 | NaHCO_3_ |

**Table S2** Sequencing depth and alpha diversity of the bacterial communities in each reservoir

| Sample ID | Reads | OTUs | Ace | Chao | Coverage, % | Shannon | Simpson | OTUs with 1 read / Total OTUs, % | OTUs with 1 read / Total reads, % |
| --- | --- | --- | --- | --- | --- | --- | --- | --- | --- |
| XJQ1 | 3853 | 313 | 1519 | 888 | 0.947054 | 3.24 | 0.0808 | 64.97 | 5.29 |
| XJQ2 | 4169 | 249 | 526 | 420 | 0.973615 | 3.73 | 0.0505 | 44.18 | 2.64 |
| XJQ3 | 3777 | 376 | 942 | 746 | 0.945989 | 3.39 | 0.1091 | 54.26 | 5.40 |
| XJL1 | 4259 | 392 | 921 | 729 | 0.956328 | 4.26 | 0.0397 | 47.45 | 4.37 |
| XJL2 | 3607 | 424 | 1188 | 806 | 0.934849 | 3.61 | 0.1214 | 55.42 | 6.52 |
| XJL3 | 4635 | 388 | 842 | 688 | 0.959655 | 3.84 | 0.0717 | 48.20 | 4.03 |
| XJL4 | 4906 | 361 | 862 | 709 | 0.961476 | 2.41 | 0.38 | 52.35 | 3.85 |
| XJL5 | 4740 | 246 | 768 | 508 | 0.97173 | 3.12 | 0.0845 | 54.47 | 2.83 |
| XJL6 | 4498 | 519 | 1269 | 915 | 0.942197 | 4.28 | 0.0552 | 50.10 | 5.78 |
| XJL7 | 3668 | 433 | 1138 | 802 | 0.937023 | 3.79 | 0.0951 | 53.35 | 6.30 |
| XJL8 | 4360 | 387 | 1137 | 818 | 0.948624 | 3.28 | 0.1094 | 57.77 | 5.11 |
| DQY1 | 3616 | 275 | 735 | 512 | 0.961283 | 3.91 | 0.0401 | 50.91 | 3.87 |
| DQY2 | 4820 | 359 | 888 | 660 | 0.9639 | 4.06 | 0.0446 | 48.47 | 3.61 |
| DQY3 | 4671 | 308 | 699 | 550 | 0.968315 | 3.25 | 0.1586 | 47.71 | 3.13 |
| DQS1 | 3418 | 252 | 807 | 483 | 0.959626 | 2.89 | 0.1869 | 54.76 | 4.04 |
| DQS2 | 3968 | 402 | 975 | 751 | 0.948337 | 3.46 | 0.1527 | 51.00 | 5.17 |
| DQS3 | 4602 | 303 | 982 | 581 | 0.961538 | 1.99 | 0.4383 | 58.42 | 3.85 |
| DQQ1 | 4736 | 297 | 744 | 533 | 0.96685 | 2.27 | 0.3691 | 52.86 | 3.32 |
| DQQ2 | 4757 | 257 | 626 | 426 | 0.974564 | 3.72 | 0.0453 | 47.08 | 2.54 |
| DQQ3 | 4252 | 258 | 776 | 544 | 0.966134 | 2.83 | 0.158 | 55.81 | 3.39 |
| DQT1 | 3852 | 436 | 1286 | 914 | 0.936137 | 3.59 | 0.1292 | 56.42 | 6.39 |
| DQT2 | 4265 | 250 | 614 | 469 | 0.970457 | 3.07 | 0.1195 | 50.20 | 2.93 |
| JL1 | 4764 | 199 | 421 | 317 | 0.980269 | 2.42 | 0.1876 | 47.24 | 1.97 |
| JL2 | 4500 | 331 | 773 | 549 | 0.962222 | 2.54 | 0.249 | 51.36 | 3.78 |
| LHS1 | 3594 | 503 | 1394 | 969 | 0.921536 | 4.06 | 0.0757 | 56.06 | 7.85 |
| LHS2 | 3453 | 473 | 1305 | 969 | 0.923545 | 3.99 | 0.0834 | 55.81 | 7.65 |
| LHJ1 | 4039 | 237 | 635 | 450 | 0.968804 | 2.51 | 0.2671 | 53.16 | 3.12 |
| LHJ2 | 5003 | 192 | 277 | 269 | 0.984409 | 1.28 | 0.6565 | 40.63 | 1.56 |
| LHD1 | 4526 | 377 | 922 | 690 | 0.956695 | 2.5 | 0.3873 | 51.86 | 4.31 |
| LHD2 | 4707 | 404 | 849 | 623 | 0.959422 | 3.05 | 0.2156 | 47.28 | 4.06 |
| SLX1 | 3149 | 282 | 823 | 507 | 0.951731 | 3.1 | 0.151 | 53.90 | 4.83 |
| SLX2 | 4787 | 226 | 837 | 541 | 0.972634 | 2.08 | 0.3602 | 57.96 | 2.74 |
| SLZ1 | 3863 | 323 | 1025 | 613 | 0.953663 | 3.41 | 0.1019 | 55.42 | 4.63 |
| SLZ2 | 3643 | 262 | 942 | 628 | 0.957178 | 2.75 | 0.1684 | 59.39 | 4.25 |
| DGD1 | 4491 | 133 | 277 | 204 | 0.986863 | 1.25 | 0.6303 | 44.36 | 1.31 |
| DGD2 | 3825 | 179 | 570 | 413 | 0.973333 | 1.94 | 0.4155 | 56.74 | 2.64 |
| DGX1 | 5728 | 241 | 346 | 322 | 0.983939 | 2.94 | 0.1255 | 38.17 | 1.61 |
| DGX2 | 3973 | 383 | 885 | 650 | 0.953687 | 3.78 | 0.0983 | 48.04 | 4.63 |
| DGX3 | 3806 | 421 | 930 | 766 | 0.942985 | 3.37 | 0.1756 | 51.54 | 5.70 |
| DGK1 | 4564 | 266 | 537 | 499 | 0.965819 | 2.07 | 0.2809 | 58.65 | 3.42 |
| DGK2 | 4405 | 274 | 646 | 545 | 0.966856 | 2.01 | 0.4164 | 53.28 | 3.31 |
| DGK3 | 4778 | 205 | 488 | 352 | 0.978652 | 1.71 | 0.4626 | 49.76 | 2.13 |
| DGS1 | 4110 | 267 | 599 | 507 | 0.96837 | 2.99 | 0.1749 | 48.69 | 3.16 |
| DGS2 | 2728 | 295 | 733 | 542 | 0.942815 | 3.68 | 0.0681 | 52.88 | 5.72 |
| YC1-1 | 4416 | 80 | 118 | 102 | 0.992754 | 0.61 | 0.8244 | 40.00 | 0.72 |
| YC1-2 | 5696 | 126 | 173 | 168 | 0.991749 | 1.28 | 0.5338 | 37.30 | 0.83 |
| YC1-3 | 4226 | 203 | 514 | 370 | 0.97539 | 2.45 | 0.2456 | 51.23 | 2.46 |
| YC2 | 4430 | 125 | 220 | 172 | 0.988939 | 1.19 | 0.6611 | 39.20 | 1.11 |
| QH1 | 3388 | 405 | 698 | 738 | 0.938902 | 3.71 | 0.0945 | 51.11 | 6.11 |
| QH2 | 4057 | 185 | 332 | 256 | 0.98102 | 2.37 | 0.2189 | 41.62 | 1.90 |
| HBM1 | 3982 | 413 | 759 | 674 | 0.955801 | 4.18 | 0.0638 | 42.62 | 4.42 |
| HBM2 | 4364 | 322 | 598 | 513 | 0.968836 | 3.66 | 0.1024 | 42.24 | 3.12 |
| HBB1 | 4743 | 304 | 612 | 470 | 0.970904 | 3.43 | 0.0799 | 45.21 | 2.89 |
| HBB2 | 4238 | 465 | 1191 | 880 | 0.943841 | 4.2 | 0.0542 | 51.18 | 5.62 |
| HBB3 | 4543 | 467 | 988 | 759 | 0.952895 | 4.1 | 0.084 | 45.82 | 4.71 |
| HBB4 | 4716 | 462 | 1196 | 845 | 0.950806 | 4.38 | 0.0291 | 50.22 | 4.92 |

**Table S3** Sequencing depth and alpha diversity of the archaeal communities in each reservoir

| Sample ID | Reads | OTUs | Ace | Chao | Coverage, % | Shannon | Simpson | OTUs with 1 read / Total OTUs, % | OTUs with 1 read / Total reads, % |
| --- | --- | --- | --- | --- | --- | --- | --- | --- | --- |
| XJQ1 | 2895 | 75 | 121 | 115 | 0.988256 | 1.27 | 0.5581 | 45.33 | 1.17 |
| XJQ2 | 2579 | 102 | 222 | 168 | 0.981388 | 1.3 | 0.6221 | 47.06 | 1.86 |
| XJQ3 | 2357 | 199 | 531 | 385 | 0.955028 | 2.96 | 0.1434 | 53.27 | 4.50 |
| XJL2 | 2226 | 190 | 498 | 374 | 0.954178 | 3.07 | 0.125 | 53.68 | 4.58 |
| XJL3 | 2199 | 206 | 515 | 390 | 0.948158 | 2.95 | 0.1415 | 55.34 | 5.18 |
| XJL4 | 2532 | 131 | 386 | 258 | 0.972354 | 2.12 | 0.3252 | 53.44 | 2.76 |
| XJL5 | 2573 | 92 | 207 | 145 | 0.982899 | 1.96 | 0.2772 | 47.83 | 1.71 |
| XJL7 | 2604 | 131 | 353 | 230 | 0.975038 | 2.54 | 0.1648 | 49.62 | 2.50 |
| XJL8 | 2372 | 113 | 292 | 219 | 0.97597 | 1.98 | 0.3551 | 50.44 | 2.40 |
| DQY1 | 2392 | 157 | 589 | 327 | 0.95903 | 1.84 | 0.3297 | 62.42 | 4.10 |
| DQY2 | 2283 | 235 | 857 | 568 | 0.933859 | 2.74 | 0.1783 | 64.26 | 6.61 |
| DQY3 | 2778 | 139 | 342 | 249 | 0.973722 | 2.07 | 0.2526 | 52.52 | 2.63 |
| DQS1 | 2440 | 236 | 694 | 466 | 0.944262 | 2.85 | 0.1651 | 57.63 | 5.57 |
| DQS2 | 2487 | 215 | 543 | 439 | 0.950945 | 2.98 | 0.1281 | 56.48 | 4.91 |
| DQS3 | 3042 | 210 | 690 | 442 | 0.957922 | 2.15 | 0.2713 | 60.95 | 4.21 |
| DQQ1 | 3026 | 154 | 766 | 418 | 0.967614 | 2.22 | 0.2481 | 63.64 | 3.24 |
| DQQ3 | 2604 | 162 | 485 | 296 | 0.96659 | 2.49 | 0.197 | 53.37 | 3.34 |
| DQT1 | 2927 | 182 | 518 | 328 | 0.96686 | 2.78 | 0.1296 | 53.30 | 3.31 |
| DQT2 | 1891 | 248 | 917 | 535 | 0.919619 | 3.14 | 0.1343 | 61.29 | 8.04 |
| JL1 | 2618 | 121 | 169 | 159 | 0.982811 | 2.75 | 0.1317 | 37.19 | 1.72 |
| JL2 | 2346 | 203 | 533 | 353 | 0.954817 | 3.24 | 0.0958 | 52.22 | 4.52 |
| LHS1 | 1806 | 151 | 451 | 299 | 0.950166 | 2.65 | 0.179 | 59.60 | 4.98 |
| LHS2 | 2243 | 252 | 611 | 446 | 0.941596 | 3.57 | 0.0773 | 51.98 | 5.84 |
| LHJ2 | 2529 | 140 | 348 | 242 | 0.97153 | 2.12 | 0.2534 | 51.43 | 2.85 |
| LHD2 | 1550 | 121 | 161 | 158 | 0.974194 | 2.8 | 0.2279 | 33.06 | 2.58 |
| SLX1 | 3173 | 216 | 614 | 475 | 0.961866 | 2.78 | 0.1272 | 56.02 | 3.81 |
| SLX2 | 2263 | 144 | 477 | 284 | 0.965091 | 2.4 | 0.2228 | 54.86 | 3.49 |
| SLZ1 | 2522 | 225 | 819 | 442 | 0.945678 | 2.65 | 0.2075 | 60.89 | 5.43 |
| SLZ2 | 2773 | 203 | 578 | 342 | 0.961774 | 2.86 | 0.207 | 52.22 | 3.82 |
| DGD1 | 2864 | 150 | 419 | 278 | 0.970321 | 1.39 | 0.6063 | 56.67 | 2.97 |
| DGX1 | 2460 | 232 | 782 | 544 | 0.945122 | 3.15 | 0.116 | 58.19 | 5.49 |
| DGX2 | 29 | 6 | 8 | 7 | 0.931034 | 1.45 | 0.2537 | 33.33 | 6.90 |
| DGX3 | 2043 | 275 | 457 | 447 | 0.9349 | 3.74 | 0.078 | 48.36 | 6.51 |
| DGK2 | 1771 | 121 | 342 | 220 | 0.963298 | 2.12 | 0.2781 | 53.72 | 3.67 |
| DGK3 | 2067 | 128 | 535 | 323 | 0.962748 | 1.93 | 0.3102 | 60.16 | 3.73 |
| DGS1 | 2441 | 133 | 358 | 248 | 0.971323 | 2.42 | 0.185 | 52.63 | 2.87 |
| DGS2 | 2743 | 150 | 553 | 329 | 0.969012 | 2.67 | 0.1648 | 56.67 | 3.10 |
| YC1-1 | 3168 | 100 | 264 | 159 | 0.984533 | 1.7 | 0.3833 | 49.50 | 1.58 |
| YC1-3 | 2256 | 94 | 166 | 171 | 0.979167 | 2.08 | 0.2146 | 50.00 | 2.08 |
| YC2 | 2695 | 130 | 329 | 219 | 0.97551 | 2.37 | 0.1693 | 50.77 | 2.45 |
| QH1 | 2699 | 137 | 224 | 230 | 0.976658 | 2.63 | 0.1524 | 45.99 | 2.33 |
| QH2 | 2783 | 69 | 145 | 95 | 0.98922 | 0.99 | 0.6862 | 43.48 | 1.08 |
| HBM1 | 1856 | 132 | 317 | 224 | 0.963901 | 2.38 | 0.2209 | 54.23 | 4.15 |
| HBM2 | 2337 | 131 | 308 | 206 | 0.972614 | 2.23 | 0.268 | 48.85 | 2.74 |
| HBB1 | 2910 | 231 | 650 | 493 | 0.955326 | 2.49 | 0.2058 | 56.28 | 4.47 |
| HBB2 | 2376 | 146 | 353 | 243 | 0.969276 | 2.1 | 0.2864 | 50.00 | 3.07 |
| HBB3 | 2844 | 157 | 326 | 268 | 0.973629 | 2.27 | 0.2786 | 47.77 | 2.64 |
| HBB4 | 2178 | 156 | 415 | 275 | 0.962351 | 2.7 | 0.1487 | 52.56 | 3.76 |

**Table S4** The dominant bacterial genera in the 22 reservoir blocks, China

|  | ALL | XJ |  | DQ |  |  |  | JL | LH |  |  | SL |  | DG |  |  |  | YC |  | QH |  | HB |  |
| --- | --- | --- | --- | --- | --- | --- | --- | --- | --- | --- | --- | --- | --- | --- | --- | --- | --- | --- | --- | --- | --- | --- | --- |
| Bacterial genera |  | XJL | XJQ | DQY | DQS | DQQ | DQT | JL | **LHD** | LHJ | LHS | SLZ | SLX | **DGX** | DGK | DGD | **DGS** | YC1 | YC2 | QH1 | QH2 | HBM | **HBB** |
|  |  | 22.6 | 39 | 44.6 | 45.7 | 50 | 60 | 65 | 38 | 47 | 60 | 65 | 66.5 | 47.5 | 60 | 66.5 | 73 | 33.9 | 39.2 | 43 | 68 | 37 | 58.4 |
| *Arcobacter* | 20.41583 | 18.75 | 2.15333 | 17.9067 | 34.7567 | 43.2733 | 0.035 | 50.79 | 1.095 | 0 | 0.14 | 0.04 | 0.05 | 3.4333 | 79.357 | 0 | 0.205 | 8.89 | 96.55 | 0.15 | 84.1 | 0.435 | 5.3725 |
| *Pseudomonas* | 8.593889 | 0.355 | 1.58667 | 5.11667 | 22.6633 | 18.7967 | 41.165 | 0.265 | 2.55 | 13.06 | 0.13 | 27.255 | 15.37 | 2.92 | 0.5233 | 3.47 | 23.57 | 0 | 0 | 0 | 0.05 | 1.175 | 0.81 |
| *Thauera* | 5.102103 | 0.225 | 5.87333 | 1.10667 | 5.61 | 2.38 | 0.7 | 0.915 | 33.87 | 0.755 | 0 | 0 | 20.18 | 0.2333 | 0.2067 | 34.37 | 0.11 | 0.0067 | 0 | 0.03 | 0 | 0.35 | 0.4475 |
| *Tepidiphilus* | 2.750833 | 0.005 | 4.52333 | 0.21667 | 0.5 | 0.04333 | 0.025 | 0 | 0.23 | 0.01 | 8.8 | 33.545 | 0.435 | 0.06 | 0 | 0 | 0.145 | 0.0067 | 0 | 0 | 0.07 | 0.115 | 9.0425 |
| *Tistrella* | 2.404603 | 0.75 | 0 | 5.13 | 0 | 0 | 0 | 0 | 0 | 0 | 0 | 0.03 | 0.03 | 0.01 | 0.0367 | 45.115 | 0.07 | 0 | 0 | 0 | 0.02 | 0 | 0.055 |
| *Acinetobacter* | 1.972341 | 0.146 | 0 | 8.63 | 0.19 | 0.03667 | 0.115 | 0 | 0.085 | 0.435 | 0 | 1.2 | 0.83 | 14.797 | 1.0733 | 0.1 | 0.565 | 0 | 0 | 1.03 | 0 | 9.99 | 2.3425 |
| ***Bacillus*** | **1.930873** | **0.016** | **0** | **0.11667** | **0.01** | **0.00667** | **0** | **0** | **0.445** | **0** | **0** | **0** | **0.03** | **33.18** | **0** | **0** | **5.315** | **0** | **0** | **0** | **0.52** | **0.135** | **0.79** |
| *Hydrogenophaga* | 1.919008 | 0.325 | 0 | 0.01333 | 0.04333 | 0 | 0.3 | 0 | 0 | 0.05 | 0.015 | 0.015 | 39.71 | 0 | 0.1 | 0 | 0.025 | 0 | 0 | 0 | 0 | 0 | 0.0325 |
| *Brevundimonas* | 1.848175 | 0.048 | 0.35333 | 0.08333 | 0 | 0.08333 | 0.855 | 0.02 | 0.01 | 0.17 | 34.96 | 0 | 0.495 | 0.2533 | 0 | 0 | 0.06 | 0.0233 | 0 | 0 | 0.02 | 1.295 | 0.13 |
| ***Lysinibacillus*** | **1.799643** | **0** | **0** | **0** | **0** | **0** | **0** | **0** | **37** | **0** | **0** | **0** | **0.065** | **0** | **0** | **0** | **0** | 0 | 0 | 0 | 0 | 0.05 | 0.6775 |
| ***Paenibacillus*** | **1.701825** | **0** | **0** | **0** | **0** | **0** | **0** | **0** | **0.51** | **0** | **0** | **0** | **0** | **0** | **0.1033** | **0** | **35.04** | 0 | 0 | 0 | 0 | 0 | 0.09 |
| *Thiomicrospira* | 1.565238 | 0 | 0 | 0 | 0 | 0 | 0 | 0 | 0 | 0 | 0 | 0 | 0 | 0 | 0 | 0 | 0 | 32.82 | 0 | 0 | 0 | 0.05 | 0 |
| *Desulfovibrio* | 1.478492 | 0.045 | 0.05667 | 0.00667 | 0.02333 | 0.01333 | 0.015 | 1.27 | 0.02 | 0.185 | 0 | 0 | 0 | 0.01 | 0.0133 | 0 | 0.02 | 0.03 | 0 | 18.27 | 10.85 | 0.015 | 0.25 |
| *Kosmotoga* | 1.385476 | 0.019 | 0.02333 | 0.00667 | 0.43333 | 0 | 0 | 0.03 | 0 | 0 | 0 | 0.015 | 0 | 0.0167 | 0 | 0 | 4.75 | 0 | 0 | 23.7 | 0.1 | 0.015 | 0.005 |
| *Dysgonomonas* | 1.249841 | 0.214 | 2.08667 | 4.79333 | 0.02667 | 0 | 0.58 | 0.01 | 0.02 | 0 | 0.955 | 0 | 2.175 | 15.24 | 0.05 | 0.205 | 0.025 | 0 | 0 | 0 | 0 | 0.055 | 0.025 |
| *Rhodobacter* | 1.131349 | 2.954 | 4.94333 | 1.06333 | 0.01 | 3.54333 | 0.91 | 0 | 0.01 | 0 | 0 | 0 | 0.015 | 0 | 12.067 | 1.085 | 0 | 0.0067 | 0 | 0 | 0.05 | 0.05 | 0.005 |
| *Rhodococcus* | 1.078611 | 0.528 | 0 | 0.45333 | 0 | 0.04333 | 0.215 | 0 | 0 | 0 | 0 | 0 | 0 | 0.1667 | 0 | 0 | 0.135 | 0 | 0.09 | 0 | 0.27 | 19.58 | 1.6975 |
| ***Anaerobacillus*** | **1.013175** | **0.008** | **0** | **0.02** | **0** | **0** | **0** | **0** | **0.02** | **0** | **0** | **0** | **0** | **0.1467** | **0** | **0** | **0.56** | **0** | **0** | **0** | **0.39** | **0.075** | **20.065** |
| *Roseovarius* | 1.012619 | 3.153 | 11.7167 | 0 | 0 | 0 | 0 | 0.01 | 0 | 0 | 0 | 0.795 | 8.38 | 0.01 | 0.2733 | 0 | 0 | 0 | 0 | 0 | 0 | 0.08 | 0 |
| *Sulfurospirillum* | 0.896706 | 1.203 | 5.67333 | 0 | 0 | 3.76333 | 4.87 | 2.815 | 0.11 | 0 | 0 | 0.75 | 0 | 0 | 0.0933 | 0 | 0 | 0.0533 | 0 | 0 | 0 | 0 | 0.7025 |
| *Oceanicola* | 0.810317 | 0 | 0 | 0 | 0 | 0 | 0 | 0 | 0 | 0 | 0 | 0 | 0 | 0 | 0 | 0 | 0 | 16.177 | 0.84 | 0 | 0 | 0 | 0 |
| *Alkalibacter* | 0.793929 | 0 | 0 | 0 | 0 | 0 | 0 | **16.66** | 0 | 0 | 0 | 0 | 0 | 0 | 0 | 0 | 0 | 0 | 0 | 0 | 0 | 0 | 0.0125 |
| *Acholeplasma* | 0.506548 | 0.526 | 0 | 0 | 0.01333 | 0 | 0.375 | 0.01 | 0.035 | 0 | 0 | 0 | 0 | 5.1767 | 0 | 4.635 | 0.01 | 0 | 0 | 0 | 0 | 0.06 | 0.3225 |
| *Oceanibaculum* | 0.47119 | 0.003 | 1.85 | 3.67667 | 0 | 0.16 | 0 | 0 | 0 | 0.11 | 0 | 3.91 | 0.015 | 0 | 0.1733 | 0 | 0 | 0 | 0 | 0 | 0 | 0 | 0 |
| *Spirochaeta* | 0.371825 | 1.35 | 0.22333 | 0 | 0.01 | 0 | 0.035 | 1.075 | 0.19 | 0 | 0 | 2.485 | 0.01 | 3.24 | 0 | 0 | 0 | 0 | 0 | 0 | 0.02 | 0 | 0.52 |
| *Thermotoga* | 0.337024 | 0 | 0 | 0.03 | 0.11667 | 0.00667 | 0.34 | 0 | 0.72 | 0 | 0.045 | 0.015 | 0 | 3.1967 | 0 | 0.01 | 0.55 | 0 | 0 | 0 | 0 | 0 | 2.0475 |
| *Rhizobium* | 0.314087 | 2.088 | 0.17 | 0.76 | 0.03333 | 0.01333 | 0.4 | 0 | 0.01 | 0.02 | 3.95 | 0 | 0 | 0.0967 | 0.0233 | 0.015 | 0.075 | 0.0067 | 0 | 0 | 0 | 0.945 | 0.0775 |
| *Paracoccus* | 0.308373 | 12.02 | 3.68667 | 0.39333 | 0 | 0.05333 | 0.245 | 0 | 0.1 | 0 | 0.03 | 0 | 0 | 0.1233 | 0.1867 | 0 | 0 | 0.29 | 0 | 0 | 0 | 0.43 | 0.9375 |
| *Sphingobium* | 0.252857 | 0.241 | 0 | 0 | 0 | 0 | 0.025 | 0 | 0.01 | 0 | 4.845 | 0.13 | 0.16 | 0 | 0 | 0.13 | 0 | 0 | 0 | 0 | 0 | 0 | 0.01 |
| *Marinomonas* | 0.249405 | 0.021 | 0 | 0 | 0 | 0 | 0 | 0 | 0 | 0 | 0 | 0 | 0 | 0 | 0 | 0 | 0 | 4.97 | 0 | 0 | 0 | 0 | 0.2675 |
| *Thermus* | 0.246071 | 0.245 | 0.10667 | 0.00667 | 0 | 0.09333 | 0.01 | 0 | 0.065 | 0 | 0.03 | 0.455 | 0 | 0.01 | 0.0067 | 0 | 0.67 | 0.0067 | 0 | 0 | 0.05 | 3.52 | 0.1375 |
| *Allobaculum* | 0.224286 | 0 | 0 | 0 | 0 | 0.01333 | 0 | 0 | 0 | 0 | 0 | 0 | 0 | 0 | 0 | 0 | 0 | 0.0067 | 0 | 0.03 | 0 | 4.66 | 0 |
| *Hyphomonas* | 0.216389 | 1.15 | 0.42 | 4 | 0 | 0 | 0.035 | 0.01 | 0 | 0 | 0 | 0 | 0 | 0.0167 | 0.0133 | 0.015 | 0 | 0.0167 | 0 | 0 | 0 | 0 | 0.0175 |
| *Dietzia* | 0.214841 | 0.098 | 4.15333 | 0 | 0 | 0 | 0.01 | 0 | 0 | 0 | 0.175 | 0 | 0.02 | 0.0167 | 0.0133 | 0.025 | 0 | 0.0233 | 0 | 0 | 0 | 0.075 | 0 |
| *Desulforhabdus* | 0.20254 | 0 | 0 | 0 | 0.02333 | 0 | 0 | 0 | 0 | 0 | 0 | 0.025 | 0 | 0 | 0 | 0 | 2.95 | 0 | 0 | 1.06 | 0 | 0 | 0.195 |
| *Microbacterium* | 0.199921 | 0.189 | 0.75 | 2.81333 | 0 | 0 | 0 | 0 | 0 | 0 | 0.29 | 0 | 0 | 0.01 | 0 | 0 | 0 | 0 | 0 | 0 | 0 | 0.315 | 0.02 |
| *Marinobacter* | 0.197302 | 0.034 | 0 | 0 | 0 | 0 | 0 | 1.135 | 0.145 | 0 | 0 | 0.155 | 0 | 0 | 0.0067 | 0 | 0.01 | 1.1167 | 0.32 | 0.06 | 0 | 0.035 | 1.16 |
| *Propionibacterium* | 0.196151 | 0.059 | 0.00667 | 0.08333 | 0 | 0.01333 | 0 | 0 | 0.01 | 0 | 0 | 0.025 | 0 | 0.3367 | 0 | 0 | 0.025 | 0.0167 | 0 | 0 | 0 | 3.275 | 0.3275 |
| *Sphingomonas* | 0.187817 | 0.076 | 0.24 | 0.02667 | 0 | 0 | 0.04 | 0.18 | 0.01 | 0 | 0.545 | 0.095 | 0 | 0.01 | 0 | 0.01 | 0.01 | 0 | 0 | 0 | 0 | 2.66 | 0.1175 |
| *Thermodesulfovibrio* | 0.18619 | 0 | 0 | 0 | 0.12667 | 0.07333 | 2.31 | 0 | 0.02 | 0 | 0 | 0 | 0 | 0.0167 | 0.0233 | 0.04 | 0.055 | 0 | 0 | 0 | 0 | 0 | 1.245 |
| *Desulfotomaculum* | 0.175238 | 0 | 0 | 0 | 0 | 0 | 0 | 0 | 0 | 0 | 0 | 0 | 0 | 0 | 0 | 0 | 0.02 | 0 | 0 | 3.66 | 0 | 0 | 0 |
| *Azospirillum* | 0.170635 | 0.003 | 0 | 1.57333 | 0 | 0 | 0 | 0 | 0.02 | 0.08 | 1.765 | 0 | 0 | 0 | 0 | 0 | 0 | 0 | 0 | 0 | 0 | 0.09 | 0.055 |
| *Bacteroides* | 0.169444 | 0.02 | 0 | 0.00667 | 0 | 0.02667 | 0 | 0 | 0 | 0 | 0 | 0 | 0.01 | 0.01 | 0 | 0 | 0 | 0 | 0 | 0 | 0 | 3.505 | 0 |
| *Marinobacterium* | 0.155635 | 0.771 | 0.15667 | 0.16667 | 0 | 0.00667 | 0 | 0.14 | 0.02 | 0 | 0 | 0 | 0 | 0 | 0.03 | 0 | 0.265 | 1.4833 | 0.54 | 0 | 0 | 0.01 | 0.45 |
| *Anoxynatronum* | 0.14123 | 0.243 | 0 | 0 | 0 | 2.61333 | 0.07 | 0.27 | 0 | 0 | 0 | 0 | 0 | 0 | 0 | 0 | 0 | 0 | 0 | 0 | 0 | 0 | 0.0125 |
| *Thermodesulfobacterium* | 0.136151 | 0 | 0 | 0 | 0 | 0 | 0 | 0 | 0 | 0.075 | 0 | 0 | 0 | 0 | 0.0067 | 0 | 0.755 | 0 | 0 | 0 | 0 | 0.71 | 1.3125 |
| *Thalassospira* | 0.129603 | 0.006 | 0 | 0 | 0 | 0 | 0 | 0 | 0 | 0 | 0 | 0 | 0 | 0 | 0 | 0.015 | 0 | 2.7067 | 0 | 0 | 0 | 0 | 0 |
| *Thermosyntropha* | 0.124127 | 0 | 0 | 0 | 0 | 0 | 0 | 0 | 0 | 0 | 0 | 0 | 0 | 0 | 0.0067 | 0 | 2.52 | 0 | 0 | 0 | 0 | 0 | 0.08 |
| *Stenotrophomonas* | 0.089008 | 0.04 | 0.06333 | 0.07667 | 0 | 0 | 0 | 0 | 0.01 | 0 | 0 | 0 | 0 | 0.06 | 0.0067 | 0.135 | 0.01 | 0 | 0 | 0.03 | 0 | 1.355 | 0.1225 |
| *Halomonas* | 0.088333 | 0.005 | 0 | 0 | 0 | 1.67333 | 0.01 | 0 | 0 | 0 | 0 | 0.165 | 0 | 0 | 0 | 0 | 0 | 0.0067 | 0 | 0 | 0 | 0 | 0 |
| *Donghicola* | 0.087262 | 12.27 | 0.40667 | 0.83667 | 0 | 0 | 0.01 | 0 | 0.11 | 0 | 0 | 0 | 0 | 0 | 0.0167 | 0 | 0 | 0 | 0 | 0 | 0.07 | 0 | 0.3825 |
| *Acetobacterium* | 0.003016 | 0.959 | 0.04333 | 0 | 0 | 0 | 0.01 | 0.01 | 0 | 0 | 0 | 0 | 0 | 0 | 0 | 0 | 0 | 0 | 0 | 0 | 0 | 0 | 0 |
| Others | 7.841911 | 9.363 | 23.3933 | 19.7467 | 4.22 | 12.4433 | 14.275 | 2.32 | 11.45 | 1.615 | 25.77 | 6.4 | 8.79 | 4.8667 | 2.1767 | 3.625 | 4.315 | 2.7667 | 1.32 | 3.87 | 2.24 | 15.56 | 9.4725 |

**Table S5** Pearson correlation coefficients of bacterial α-diversity metrics and population abundance with the reservoir physicochemical parameters

|  | **Temperature** | **pH** | **Salinity** | **SO_4_^2-^** | **Ca^2+^** | **Mg^2+^** |
| --- | --- | --- | --- | --- | --- | --- |
| Shannon diversity | -.147 | **.400^**^** | -.149 | **-.448^**^** | **-.393^**^** | **-.342^**^** |
| Simpson diversity | .047 | **-.303^*^** | .103 | **.502^**^** | **.389^**^** | **.374^**^** |
| *Arcobacter* | .046 | -.043 | .169 | .026 | .000 | **.395^**^** |
| *Pseudomonas* | **.279^*^** | **-.380^**^** | -.066 | .025 | -.131 | -.102 |
| *Paracoccus* | **-.613^**^** | **.664^**^** | -.059 | -.128 | -.104 | -.080 |
| *Thiomicrospira* | -.127 | -.176 | .197 | **.658^**^** | **.566^**^** | .085 |
| *Donghicola* | **-.437^**^** | **.484^**^** | -.035 | -.073 | -.070 | -.048 |
| *Desulfovibrio* | .071 | .050 | **.860^**^** | -.010 | **.334^*^** | .215 |
| *Kosmotoga* | .040 | .008 | **.697^**^** | .038 | .226 | .076 |
| *Deferribacteraceae*_uncultured | .178 | -.140 | -.055 | **.317^*^** | -.078 | -.051 |
| *Rhizobium* | -.206 | **.295^*^** | -.091 | -.093 | -.084 | -.058 |
| *Hyphomonas* | **-.263^*^** | **.288^*^** | -.089 | -.091 | -.092 | -.054 |
| *Marinomonas* | -.123 | -.170 | .154 | .132 | **.505^**^** | .081 |
| *Marinobacterium* | **-.305^*^** | .147 | .097 | .071 | **.330^*^** | .108 |
| *Comamonas* | .096 | **-.269^*^** | -.015 | .000 | -.063 | -.053 |
| *Thalassospira* | -.128 | -.175 | .160 | .136 | **.508^**^** | .083 |
| *Acetobacterium* | -.261 | **.286^*^** | -.031 | -.051 | -.043 | -.027 |
| *Methylobacterium* | -.255 | **.266^*^** | -.041 | -.065 | -.054 | -.038 |
| *Azonexus* | .117 | **-.279^*^** | .000 | -.030 | -.050 | -.045 |
| *Desulfotomaculum* | -.043 | .037 | **.705^**^** | .026 | .249 | .088 |
| *Propionicimonas* | **-.391^**^** | **.446^**^** | -.031 | -.079 | -.067 | -.051 |
| *Geoalkalibacter* | -.073 | -.197 | .141 | .164 | **.399^**^** | .249 |
| *Ruminococcaceae*_uncultured | **-.289^*^** | .282^*^ | -.061 | -.067 | -.072 | -.052 |
| *Dethiosulfatibacter* | .162 | .070 | **.443^**^** | -.089 | .190 | .226 |
| *Acidaminobacter* | **-.265^*^** | .291^*^ | -.017 | -.043 | -.045 | -.033 |
| *Eubacteriaceae*_uncultured | .102 | **-.282^*^** | .015 | -.021 | -.042 | -.039 |
| *Christensenellaceae*_uncultured | **-.340^*^** | **.443^**^** | -.069 | -.102 | -.095 | -.069 |
| *Longilinea* | **-.324^*^** | **.347^**^** | -.030 | -.057 | -.053 | -.047 |
| *Coriobacteriaceae*_uncultured | -.246 | **.321^*^** | -.063 | -.082 | -.077 | -.059 |
| *Hydrogenophilus* | **.282^*^** | -.092 | .130 | .031 | -.026 | -.022 |
| *Guggenheimella* | **-.344^**^** | **.351^**^** | .107 | .261 | -.044 | **.665^**^** |
| *Hydrocarboniphaga* | .239 | -.181 | -.044 | **.454^**^** | -.058 | -.033 |
| *Novosphingobium* | **-.283^*^** | **.277^*^** | -.031 | -.078 | -.070 | -.053 |
| *Methylophilus* | -.280^*^ | **.311^*^** | -.026 | -.066 | -.054 | -.036 |
| *Halocella* | -.045 | .038 | **.704^**^** | .026 | .249 | .088 |
| *Marinitoga* | -.109 | -.131 | .245 | **.370^**^** | .411^**^ | **.615^**^** |
| *Sphingopyxis* | **-.267^*^** | .190 | .025 | .090 | -.074 | **.294^*^** |
| *Castellaniella* | -.298^*^ | **.333^*^** | -.024 | -.059 | -.052 | -.042 |
| *Desulfuromonas* | **-.331^*^** | **.424^**^** | -.066 | -.083 | -.077 | -.061 |
| *Rhodobacteraceae*_uncultured | -.249 | **.360^**^** | -.087 | -.109 | -.100 | -.079 |
| SC103 | .123 | .098 | **.480^**^** | -.058 | .233 | **.273^*^** |
| *Desulfosarcina* | -.045 | .038 | **.704^**^** | .026 | .249 | .088 |
| *Anaerolinea* | **-.347^**^** | **.450^**^** | -.060 | -.086 | -.083 | -.067 |
| *Belliella* | .151 | **-.292^*^** | -.001 | -.031 | -.053 | -.047 |
| *Pelobacter* | **-.278^*^** | **.369^**^** | -.063 | -.072 | -.066 | -.056 |
| *Ruminococcaceae_Incertae_Sedis* | -.040 | .070 | **.537^**^** | -.020 | .169 | .040 |
| *Dokdonella* | .108 | **-.283^*^** | .002 | -.025 | -.042 | -.038 |
| *Blastomonas* | **-.273^*^** | **.301^*^** | -.014 | -.056 | -.045 | -.032 |
| *Marinilabilia* | .138 | -.012 | **.518^**^** | -.011 | **.376^**^** | .297^*^ |
| *Pseudobutyrivibrio* | .180 | .038 | **.490^**^** | -.051 | .244 | .283^*^ |

N=56. *Correlation is significant at the 0.05 level; **Correlation is significant at the 0.01 level

**Table S6** Pearson correlation coefficients of archaeal α-diversity metrics and population abundance with the reservoir physicochemical parameters

|  | **Temperature** | **pH** | **Salinity** | **SO_4_^2-^** | **Ca^2+^** | **Mg^2+^** |
| --- | --- | --- | --- | --- | --- | --- |
| Shannon diversity | .108 | -.069 | -.176 | -.083 | -.270 | -.151 |
| Simpson diversity | -.051 | .095 | .165 | -.033 | .206 | .084 |
| *Methanothermobacter* | **.495^**^** | **-.349^*^** | -.208 | -.005 | -.206 | -.168 |
| *Methanolinea* | **-.375^**^** | .282 | -.140 | -.141 | -.125 | -.095 |
| *Methanothermococcus* | .016 | -.108 | **.683^**^** | **.439^**^** | **.777^**^** | **.486^**^** |
| *Methanococcus* | **-.305^*^** | .156 | .059 | .054 | .114 | .149 |
| *Methanocorpusculum* | **-.490^**^** | **.536^**^** | -.028 | -.081 | -.066 | -.054 |
| *Thermococcus* | **.507^**^** | -.247 | .060 | .243 | -.082 | -.060 |
| *Methanosarcina* | -.102 | .007 | -.014 | .172 | .065 | **.322^*^** |
| *Halogranum* | **.307^*^** | -.101 | .065 | .022 | -.029 | -.033 |

N=48. *Correlation is significant at the 0.05 level; **Correlation is significant at the 0.01 level

**Table S7** One-Way ANOVA of the abundance of bacterial classes between the oilfields, reservoirs, reservoirs within the same oilfield, and temperatures

| Taxa | P value | | | | | | | | | |
| --- | --- | --- | --- | --- | --- | --- | --- | --- | --- | --- |
|  | Oilfields | Reservoirs | XJ | DQ | LH | SL | DG | HB | Temp | Temp-DQ |
| *Epsilonproteobacteria* | .111 | .007 | .458 | .597 | .498 | .445 | .001 | .397 | .340 | .439 |
| *Alphaproteobacteria* | .015 | .021 | .717 | .008 | .001 | .587 | .353 | .008 | .048 | .469 |
| *Betaproteobacteria* | .051 | .122 | .281 | .382 | .520 | .544 | .307 | .505 | .060 | .339 |
| *Clostridia* | .001 | .020 | .228 | .529 | .012 | .279 | .536 | .734 | .749 | .457 |
| *Actinobacteria* | .113 | .001 | .183 | .417 | .006 | .337 | .575 | .037 | .034 | .380 |
| *Deltaproteobacteria* | .000 | .000 | .331 | .596 | .927 | .168 | .296 | .214 | .725 | .884 |
| *Bacteroidia* | .935 | .746 | .032 | .032 | .512 | .465 | .591 | .180 | .722 | .808 |
| *Thermotogae* | .004 | .000 | .946 | .260 | .503 | .423 | .432 | .354 | .509 | .613 |
| *Deferribacteres* | .049 | .144 | .035 | .416 |  | .701 | .288 | .343 | .026 | .628 |
| *Anaerolineae* | .159 | .246 | .099 | .042 | .117 | .423 | .641 | .137 | .271 | .088 |
| *Thermoleophilia* | .309 | .032 |  | .510 | .392 |  |  | .447 | .726 | .289 |
| *Planctomycetacia* | .477 | .122 | .691 | .338 | .453 |  | .586 | .019 | .844 | .610 |
| *Ignavibacteria* | .435 | .000 | .053 | .000 | .650 |  |  | .462 | .327 | .281 |
| *Flavobacteria* | .086 | .204 | .527 | .002 |  |  | .623 | .179 | .449 | .000 |
| *Thermodesulfobacteria* | .001 | .090 |  |  | .465 |  | .226 | .618 | .417 |  |
| *VC2.1_Bac22* | .317 | .918 | .337 |  | .465 |  |  | .417 | .044 |  |
| *Acidimicrobiia* | .172 | .001 | .256 |  | .245 |  |  | .523 | .558 |  |
| *Nitriliruptoria* | .000 | .121 |  |  |  |  |  |  | .308 |  |
| *Caldilineae* | .122 | .000 |  | .103 | .054 |  |  |  | .560 | .460 |

**Table S8** One-Way ANOVA of the abundance of bacterial classes between the oilfields, reservoirs, reservoirs within the same oilfield, and temperatures

| Taxa | P value | | | | | | | | | |
| --- | --- | --- | --- | --- | --- | --- | --- | --- | --- | --- |
|  | Oilfields | Reservoirs | XJ | DQ | LH | SL | DG | HB | Temp | Temp-DQ |
| *Arcobacter* | .233 | .001 | .259 | .581 | .502 | .890 | .001 | .411 | .630 | .422 |
| *Paracoccus* | .000 | .000 | .090 | .562 | .406 |  | .465 | .560 | .000 | .825 |
| *Rhodobacter* | .789 | .863 | .594 | .631 | .465 | .423 | .601 | .035 | .812 | .431 |
| *Brevundimonas* | .021 | .000 | .158 | .270 | .001 | .399 | .478 | .005 | .293 | .125 |
| *Donghicola* | .046 | .456 | .220 | .512 | .465 |  | .586 | .313 | .001 | .711 |
| *Dysgonomonas* | .896 | .715 | .009 | .000 | .471 | .388 | .590 | .533 | .627 | .294 |
| *Sulfurospirillum* | .127 | .027 | .051 | .112 | .465 | .423 | .586 | .359 | .096 | .040 |
| *Rhodococcus* | .011 | .000 | .427 | .401 |  |  | .641 | .038 | .155 | .777 |
| *Oceanicola* | .082 | .738 |  |  |  |  |  |  | .049 |  |
| *Phenylobacterium* | .916 | .589 | .106 | .293 | .473 | .423 | .013 | .178 | .693 | .600 |
| *Desulfovibrio* | .000 | .000 | .813 | .809 | .496 |  | .796 | .443 | .774 | .993 |
| *Kosmotoga* | .000 | .000 | .896 | .244 |  | .423 | .269 | .447 | .770 | .548 |
| *Deferribacteraceae_uncultured* | .005 | .099 | .034 | .387 |  | .703 | .285 | .288 | .324 | .654 |
| *Alkalibacter* | .000 | .110 |  |  |  |  |  | .541 | .300 |  |
| *Rhizobium* | .744 | .953 | .496 | .273 | .421 |  | .733 | .040 | .503 | .606 |
| *Oceanibaculum* | .401 | .018 | .104 | .034 | .465 | .315 | .427 |  | .575 | .408 |
| *Jonesia* | .767 | .388 |  | .370 |  |  | .285 |  | .000 | .185 |
| *Hyphomonas* | .425 | .051 | .611 | .002 |  |  | .868 | .541 | .333 | .288 |
| *Dietzia* | .809 | .682 | .100 | .217 | .246 | .423 | .805 | .000 | .404 | .092 |
| *Patulibacter* | .324 | .041 |  |  | .403 |  |  | .447 | .735 |  |
| *Sphingobium* | .306 | .018 | .221 | .217 | .358 | .898 | .285 | .541 | .737 | .092 |
| *Microbacterium* | .660 | .039 | .256 | .121 | .338 |  | .586 | .000 | .613 | .476 |
| *Ignavibacterium* | .066 | .000 | .485 | .000 | .465 |  |  | .462 | .146 | .282 |
| *Propionibacterium* | .028 | .000 | .549 | .549 | .465 | .423 | .600 | .080 | .265 | .783 |
| *Anoxynatronum* | .895 | .792 | .510 | .515 |  |  |  | .541 | .011 | .293 |
| *Sphingomonas* | .024 | .000 | .310 | .471 | .438 | .423 | .745 | .002 | .028 | .441 |
| *Azospirillum* | .345 | .001 | .568 | .103 | .150 |  |  | .644 | .598 | .460 |
| *Thermodesulfobacterium* | .001 | .090 |  |  | .465 |  | .226 | .618 | .417 |  |
| *Serratia* | .018 | .143 | .518 | .510 |  |  | .586 | .313 | .322 | .289 |
| *EscherichiaShigella* | .002 | .162 | .320 | .510 |  |  | .586 | .448 | .379 | .706 |
| *Methylobacterium* | .861 | .999 | .557 | .604 | .533 | .423 |  | .000 | .557 | .409 |
| *Desulfobacula* | .008 | .046 | .391 |  | .465 |  | .285 | .214 | .359 |  |
| *Synergistaceae_uncultured* | .872 | .806 | .972 | .533 | .465 | .423 | .586 | .541 | .013 | .310 |

**Table S9** One-Way ANOVA of the abundance of bacterial classes between the oilfields, reservoirs, reservoirs within the same oilfield, and temperatures

| Taxa | P value | | | | | | | | | |
| --- | --- | --- | --- | --- | --- | --- | --- | --- | --- | --- |
|  | Oilfields | Reservoirs | XJ | DQ | LH | SL | DG | HB | Temp | Temp-DQ |
| *Methanomicrobia* | .036 | .017 | .863 | .051 | .302 | .642 | .177 | .028 | .025 | .018 |
| *Methanobacteria* | .283 | .126 | .736 | .046 | .356 | .972 | .281 | .381 | .172 | .016 |
| *Methanococci* | .000 | .001 | .395 | .632 | .012 | .423 | .529 | .541 | .048 | .695 |
| *Archaeoglobi* | .246 | .420 |  | .044 | .816 | .340 | .495 | .328 | .200 | .012 |
| *Thermococci* | .013 | .000 |  | .041 | .865 | .221 | .021 | .440 | .000 | .011 |
| *Thermoprotei* | .239 | .490 |  | .382 | .620 | .347 | .595 | .658 | .026 | .216 |
| *Thermoplasmata* | .001 | .000 | .133 | .079 | .009 | .211 | .528 | .072 | .883 | .115 |
| *Halobacteria* | .014 | .000 | .731 | .285 |  | .653 | .760 | .541 | .590 | .128 |

**Table S10** One-Way ANOVA of the abundance of bacterial classes between the oilfields, reservoirs, reservoirs within the same oilfield, and temperatures

| Taxa | P value | | | | | | | | | |
| --- | --- | --- | --- | --- | --- | --- | --- | --- | --- | --- |
|  | Oilfields | Reservoirs | XJ | DQ | LH | SL | DG | HB | Temp | Temp-DQ |
| *Methanothermobacter* | .021 | .002 | .316 | .008 | .395 | .382 | .286 | .329 | .004 | .002 |
| *Methanosaeta* | .033 | .002 | .535 | .063 | .278 | .555 | .005 | .947 | .101 | .036 |
| *Archaeoglobus* | .192 | .457 |  | .075 | .816 | .982 | .563 | .336 | .391 | .023 |
| *Methanothermococcus* | .000 | .000 | .516 |  |  | .423 | .497 |  | .036 |  |
| *Methanococcus* | .184 | .904 | .393 | .630 | .019 | .423 | .691 |  | .220 | .683 |
| *Methanoculleus* | .174 | .898 | .606 | .522 |  | .525 | .698 | .025 | .365 | .428 |
| *Methanocorpusculum* | .035 | .213 | .127 | .267 |  | .576 | .751 | .541 | .000 | .612 |
| *Methanocalculus* | .177 | .023 | .389 | .285 |  | .521 | .319 | .541 | .584 | .128 |
| *Methanocella* | .090 | .000 | .316 | .531 |  | .423 | .479 | .005 | .056 | .446 |
| *Thermococcus* | .027 | .000 |  | .041 | .865 | .534 | .030 | .440 | .000 | .011 |
| *Methanofollis* | .000 | .000 | .516 | .227 | .816 | .423 | .751 | .178 | .032 | .095 |
| *Candidatus_Nitrososphaera* | .194 | .000 |  |  |  |  | .479 |  | .023 |  |
| *Methanosarcina* | .117 | .000 | .320 |  |  | .445 | .479 |  | .006 |  |
| *Candidatus_Nitrosotalea* | .193 |  |  |  |  |  |  |  | .023 |  |
| *Geoglobus* | .236 | .311 |  |  | .816 | .423 | .030 |  | .800 |  |
| *Halogranum* | .710 | .370 |  |  |  | .423 | .479 |  | .012 |  |
